# Characteristics of Antibiotic Resistance Genes and Antibiotic-Resistant Bacteria in Full-Scale Drinking Water Treatment System Using Metagenomics and Culturing

**DOI:** 10.3389/fmicb.2021.798442

**Published:** 2022-02-22

**Authors:** Qihui Gu, Ming Sun, Tao Lin, Youxiong Zhang, Xianhu Wei, Shi Wu, Shuhong Zhang, Rui Pang, Juan Wang, Yu Ding, Zhenjie Liu, Ling Chen, Wei Chen, Xiuhua Lin, Jumei Zhang, Moutong Chen, Liang Xue, Qingping Wu

**Affiliations:** Guangdong Provincial Key Laboratory of Microbial Safety and Health, State Key Laboratory of Applied Microbiology Southern China, Institute of Microbiology, Guangdong Academy of Sciences, Guangzhou, China

**Keywords:** antibiotic resistance genes, antibiotic resistant bacteria, metagenomic approach, metagenome-assembled genomes, pathogens, DWTS

## Abstract

The contamination of antibiotic resistance genes (ARGs) may directly threaten human health. This study used a metagenomic approach to investigate the ARG profile in a drinking water treatment system (DWTS) in south China. In total, 317 ARG subtypes were detected; specifically, genes encoding bacitracin, multidrug, and sulfonamide were widely detected in the DWTS. Putative ARG hosts included *Acidovorax* (6.0%), *Polynucleobacter* (4.3%), *Pseudomonas* (3.4%), *Escherichia* (1.7%), and *Klebsiella* (1.5%) as the enriched biomarkers in the DWTS, which mainly carried bacitracin, beta-lactam, and aminoglycoside ARGs. From a further analysis of ARG-carrying contigs (ACCs), *Stenotrophomonas maltophilia* and *Pseudomonas aeruginosa* were the most common pathogens among the 49 ACC pathogens in the DWTS. The metagenomic binning results demonstrated that 33 high-quality metagenome-assembled genomes (MAGs) were discovered in the DWTS; particularly, the MAG identified as *S. maltophilia*-like (bin.195) harbored the greatest number of ARG subtypes (*n* = 8), namely, multidrug (*n* = 6; *sme*D, *sem*E, multidrug_transporter, *mex*E, *sem*B, and *sme*C), beta-lactam (*n* = 1; metallo-beta-lactamase), and aminoglycoside [*n* = 1; *aph*(3’)-IIb]. The strong positive correlation between MGEs and ARG subtypes revealed a high ARG dissemination risk in the DWTS. Based on the pure-culture method, 93 isolates that belong to 30 genera were recovered from the DWTS. Specifically, multidrug-resistant pathogens and opportunistic pathogens such as *P. aeruginosa*, *Bacillus cereus*, and *S. maltophilia* were detected in the DWTS. These insights into the DWTS’s antibiotic resistome indicated the need for more comprehensive ARG monitoring and management in the DWTS. Furthermore, more effective disinfection methods need to be developed to remove ARGs in DWTSs, and these findings could assist governing bodies in the surveillance of antibiotic resistance in DWTSs.

## Introduction

Antibiotic resistance genes (ARGs) are emerging environmental pollutants (Pruden et al., 2006). The excessive use and abuse of antibiotics in medicine and agriculture have promoted the emergence, spread, and accumulation of ARGs in various environmental matrices (Jia et al., 2019), including natural and man-impacted environments, such as soil (Fang et al., 2015), sediment (Jiang et al., 2018), surface water (Zhang et al., 2020b), wastewater (Wang et al., 2020), groundwater (Wu et al., 2020), drinking water (Wan et al., 2020), and tap water (Ma et al., 2019). Thus, antimicrobial resistance has become a global issue and is regarded as a medical concern by both researchers and the public.

Additionally, antibiotic-resistant bacteria (ARB) have become a serious threat to human health. In particular, pathogenic bacteria might directly affect the treatability of patients with infectious diseases (Kumar et al., 2019). There is some literature on the occurrence of ARB in drinking water and the evaluation of the impact of ARB on human health. However, most of the research has only investigated one species of bacteria or a group of resistant bacteria, such as *Pseudomonas aeruginosa* strains (Mombini et al., 2019), aminoglycoside-resistant bacteria (Ullmann et al., 2019), *Escherichia coli*, and *Salmonella* spp. (Perez-Vidal et al., 2019), and the presence of β-lactamase-producing Gram-negative bacteria (Torkar and Drazetic, 2017). There has been limited comprehensive research on the antibiotic resistance of different species of bacteria or multigroup-resistant bacteria in a drinking water treatment system (DWTS). Thus, a comprehensive risk identification of ARB in DWTSs has not been conducted.

Thus, it is necessary to fully understand the occurrence and abundance of ARGs and ARB in DWTSs. However, very few studies have reported the occurrence and distribution of ARGs and ARB in the whole drinking water treatment process. Bergeron et al. investigated the ARB and ARGs in raw source water and treated drinking water and found that the ARGs of sulfonamides and tetracycline antibiotics and ARB were frequently observed in the source water (Bergeron et al., 2015). Moreover, Bergeron et al. surveyed the presence of ARGs and ARB in a rural drinking water plant in Louisiana, United States, and demonstrated that ARGs and ARB were present in the raw intake water but not in the treated and distributed water (Bergeron et al., 2017). With the development of sequencing technology and high-throughput DNA testing techniques, more ARGs have been discovered in finished water and tap water, which arouses great concern as a public health issue. For example, Su et al. used the quantitative PCR (qPCR) method to investigate the occurrence and diversity of ARGs in the DWTS and found that most ARGs, including *sul*1, *erm*B, *tet*Q, *tet*W, *cfr*, *cml*A, *fex*A, *fex*B, *flo*R, and *qnr*S, significantly increased from finished water in the distribution system to tap water, and *Pseudomonas* might be involved in the proliferation and spread of ARGs in the DWTS (Su et al., 2018). Furthermore, Xu et al. used high-throughput qPCR to detect ARGs in the DWTS, and the results demonstrated that 285 ARGs were discovered, which provided comprehensive information on the prevalence of ARGs in the DWTS (Xu et al., 2016).

Compared to qPCR and high-throughput qPCR approaches, a high-throughput sequencing-based metagenomic analysis can overcome the drawbacks of amplification-based methods, including the limited availability of primers, the possible bias in the amplification process, and the false-negative results caused by an enzyme inhibitor in the environmental samples. Moreover, a metagenomic approach could obtain more comprehensive profiles of ARGs. For example, Bai et al. applied metagenomic sequencing to investigate the prevalence of ARGs in drinking water sources, and the results indicated that 71 ARG subtypes within 14 ARG types were present (Bai et al., 2019). Jia et al. also applied metagenomic sequencing to investigate the changing profiles of ARGs and bacterial community in a DWTS and demonstrated that 151 ARG subtypes within 15 ARG types were detected, and chlorination increased their total relative abundance and reduced their diversity in the opportunistic bacteria (Jia et al., 2015). Therefore, a metagenomic approach could assist in investigating the full profile of ARGs in the DWTS comprehensively and determining ARB biomarkers as putative ARG carriers.

Mobile genetic elements (MGEs) play a key role in transferring ARGs among different microorganisms in the environments (Zhu et al., 2019). MGEs (e.g., plasmids, transposons, and integrons) are widely distributed in environments, and they help the dissemination of ARGs *via* horizontal gene transfer (Zhang et al., 2019). Statistics and network analyses have demonstrated a close positive correlation between ARG subtypes and MGEs (Qian et al., 2018; Zeng et al., 2019; Pu et al., 2020). Moreover, recently, a metagenomic analysis provided direct evidence on the co-occurrence patterns of ARGs and MGEs, which accurately indicated the possible transfer potential of ARGs in environmental compartments such as feces, soils, and water (Zhang et al., 2020c).

The Pearl River Delta (PRD) is a densely populated area, and the three major tributaries of the Pearl River Basin—Dongjiang, Xijiang, and Beijiang—are the main drinking water sources, serving more than 1.4 million people in Guangzhou, China (Su et al., 2018). However, information about the presence and abundance of ARGs, MGEs, and ARB in the DWTS in this area is still limited. Although some researchers have investigated the ARG profiles of drinking water sources in the PRD region using a metagenomic approach, the occurrence and abundance of ARGs in different phases of the DWTS in the PRD region have not been determined (Jiang et al., 2018). Additionally, researchers have investigated the limited target ARGs in the DWTS in the PRD region, but there has been no comprehensive research on MGEs and ARB in the studied area (Su et al., 2018). Subsequently, the results of the present study can provide a better understanding of the diversity, abundance, and dissemination of ARGs and ARB in the DWTS in the PRD region.

## Materials and Methods

### Sample Collection and DNA Extraction

**FIGURE 1:**
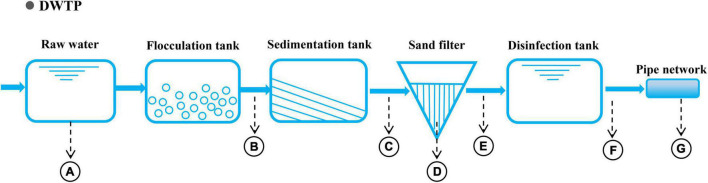
Process diagram of the DWTP and sampling sites. Sampling points: A: raw water; B: flocculation tank effluent; C: sedimentation tank effluent; D: quartz sand; E: sand filter effluent; F: chlorine-disinfected water; and G: tap water.

The raw water from the selected drinking water treatment plant (DWTP) (Guangzhou, China) originates in the North Rivers of Pearl River, and its phased treatment includes flocculation, sedimentation, filtration, and chlorination (Figure 1). Water samples were collected along the treatment processes of the DWTP and distribution pipeline, including samples for raw water (A), flocculation tank effluent (B), sedimentation tank effluent (C), quartz sand (D), sand filter effluent (E), chlorine-disinfected water (F), and tap water (G). They were respectively collected from a water pumping station (5 L), flocculation tank (18 L), sedimentation tank (18 L), sand filter (36 L), clear water tank (54 L), and tap water (about 2,000 L) (Figure 1). Additionally, quartz sand samples (D) were obtained from the upper part of the quartz-sand layer in the sand filter (50 g). To minimize the temporal variation, three replicate water samples were simultaneously collected from each location (21 samples in total) in July 2019. All of the samples were immediately transported to a laboratory for the experiments. The water sampling and pre-treatment methods were performed as previously described^27^. DNA for each sample was extracted using a FastDNA SPIN Kit for Soil (MP Biomedicals, CA, United States), and the DNA concentration and purity were measured using a microspectrophotometry (Nano DropND-2000, NanoDrop Technologies, Wilmington, DE, United States).

### Water Quality Analyses

For each sampling site, temperature, pH, and dissolved oxygen (DO) level were measured *in situ* using an HQ40D portable multi-parameter water quality analyzer (HACH, Loveland, CO, United States). Turbidity was measured using a 2100Q portable turbidimeter (HACH, Loveland, CO, United States). Ammonia-nitrogen, chemical oxygen demand (COD), and total organic carbon (TOC) were measured using a DR1900 portable spectrophotometer (HACH, Loveland, CO, United States). Adenosine triphosphate (ATP) level was measured with an ATP assay kit (Beyotime, Shanghai, China). All determinations were performed in triplicate.

### Cultivation and Molecular Identification of Bacteria

Bacteria were isolated from the water samples of the different sampling sites. Briefly, 0.2 ml of water samples or proper dilution of the water samples was spread on R2A agar plates with an L-type coating rod. Then, all the plates were incubated at 22°C for 7 days. Subsequently, total heterotrophic bacterial counts (HPCs) were determined by counting the number of colonies on the R2A agar plates. Next, colonies with different morphologies were picked and purified using the streaking method (Massa et al., 1998). The process was repeated until a pure bacterial strain was obtained. To identify the obtained bacteria, all pure isolates were first incubated in R2A broth for 24 h, and then, genomic DNA for each pure isolate was extracted using a DNA extraction kit (Magen Biotech, Guangzhou, China). Subsequently, the 16S rRNA gene was amplified in a 25-μl volume using universal primers 27 F and1492 R (Mas-Ud et al., 2020), and amplification was performed as previously described (Gu et al., 2018). Then, PCR products were sequenced by BGI Tech Solutions Co., Ltd (Guangzhou, China), and 16S rRNA gene sequences were submitted to the NCBI public library of bioinformatics for comparison and identification.^1^

### Antimicrobial Susceptibility Testing

Antibiotic resistance of the representative bacterial isolates from the DWTS was determined using the standard Kirby–Bauer disc diffusion method according to Clinical and Laboratory Standards Institute Guidelines (CLSI, 2020). The antibiotics and concentrations of the disks (Oxoid, Basingstoke, United Kingdom) used in this study were as follows: piperacillin-tazobactam (TZP, 100/10 μg), norfloxacin (NOR, 10 μg), clindamycin (DA, 2 μg), cefepime (FEP, 3 μg), ceftriaxone (CRO, 30 μg), ciprofloxacin (CIP, 5 μg), penicillin (P, 10 μg), cefotaxime (CTX, 30 μg), chloramphenicol (C, 30 μg), ampicillin (AMP, 10 μg), trimethoprim-sulfamethoxazole (SXT, 1.25/23.75 μg), erythromycin (E, 15 μg), aztreonam (ATM, 30 μg), meropenem (MEM, 10 μg), cefoxitin (FOX, 30 μg), amikacin (AK, 30 μg), kanamycin (K, 30 μg), tobramycin (TOB, 10 μg), amoxicillin-clavulanate (AMC, 30 μg), tetracycline (TE, 30 μg), gentamicin (CN, 10 μg), ceftazidime (CAZ, 30 μg), and levofloxacia (LEV, 5 μg). Additionally, multiple antibiotic resistance index (MARI) values were calculated for all the isolates to evaluate whether they originated from high-risk antibiotic pollution sources (Labella et al., 2021).

### Metagenomic Sequencing and Metagenomic Analysis

The extracted DNA from each sample was sequenced using the Illumina HiSeq 2500 platform (Illumina Inc., San Diego, CA, United States). Over 10 Gb of raw data was generated for each sample (Supplementary Table 1). Quality control and assembly were conducted as described previously (Bai et al., 2021). To obtain low-abundance species, the obtained clean data were mapped to its own Scaftigs to obtain un-using reads, and then all the un-using reads were collated for hybrid packaging. The open reading frames (ORFs) within the contigs of each sample were subsequently predicted using Prodigal.^2^ The gene prediction results were used for gene clustering by Linclust.^3^ The longest sequence in each cluster was taken as the representative sequence to obtain the non-redundant gene catalogue (Unigenes). Clean reads were mapped to the gene catalogue by BBmap,^4^ and the relative abundance of each gene in each sample was calculated using the number of reads mapped and gene length with the following formula:

$$G_{k}=\frac{r_{k}}{L_{k}}\times \frac{1}{\sum_{i=1}^{n}\frac{r_{i}}{L_{i}}}$$

where k stands for any identified gene, G_k_ is the relative abundance of each identified gene, r_k_ is the number of mapped metagenomic reads for each identified gene, and L_k_ is the length of each identified gene. ARGs were detected against a structured ARG reference database (SARG version 2.0) with the following parameters: under a cutoff of ≥80% similarity and ≥70% query coverage (Yin et al., 2018). Furthermore, an online analysis pipeline (ARGs-OAP) based on the SARG database was used for the ARG-like sequence annotation from the metagenomic sequencing reads with default settings (Yin et al., 2018). Subsequently, the abundance of ARGs was normalized and expressed as the number of copies of ARGs per cell (copy/cell) (Ma et al., 2017). A contig was retrieved as an ARG-carrying contig (ACC) if it contained ≥ one ARG-ORF (Zhang et al., 2019). The contigs that harbored ARGs were selected, and the protein sequences on the contigs were used to make a comparison against the local NCBI NR database using BLASTP with a cutoff of E-value ≤ 10^–5^ (Mao et al., 2014). Subsequently, the MEtaGenome ANalyzer (MEGAN, version 6) was used to align the profiles. If ≥ 50% of ORFs on a contig were attributed to the same taxon, these ARG-carrying contigs (ACCs) were assigned to this taxon (Ishii et al., 2013). ARG-carrying pathogens were determined according to a previous study (Li et al., 2015).

The plasmids, integrons, and insert sequences were identified based on aclame, integration, and ISfinder databases with the following parameters: under a cutoff of identity ≥80% and query cover ≥70%. Metagenome binning, bin checking, and taxonomic annotation were performed as previously described (Poghosyan et al., 2020). The metagenome bins that were >50% complete with <10% contamination were retained for building the phylogenomic tree using GToTree (https://github.com/AstrobioMike/GToTree/wiki). The abundance [reported as reads per kilobase per million (RPKM)] of genes was conducted as previously described (Potgieter et al., 2020). The occurrence of ARGs in chromosomes or plasmids was also determined as previously described (Camacho et al., 2009), and MAFFT (Katoh and Standley, 2013) was used to align the amino acid sequences of *IntI* genes recovered from the metagenomes together with the reference sequences of class 1 (*IntI*1, AAQ16665.1) integrases. Raw data have been submitted to the NCBI Sequence Read Archive (SRA) under accession number SRR17063149-17063169.

## Results and Discussion

### Water Quality

The results of the physicochemical and microbiological parameters of water samples are illustrated in Table 1. The water temperature was 27–28°C, and the water pH was 7.63–8.16. The turbidity (NTU) decreased significantly from 69.47 to 2.62 NTU after the settling process. Water DO maintained a steady state during the water treatment process. However, the ATP and HPC gradually decreased. After chlorination, no cultivable bacteria were detected. However, in the tap water, 10 CFU/ml HPC were detected. COD_Mn_ decreased gradually with the treatment process and increased slightly after entering the pipe network. TOC was always kept at a very low level. Other parameters, such as TN, also demonstrated a downward trend. They remained at a low concentration in the chlorine-disinfected water, even in the tap water. All the physicochemical qualities of the chlorine-disinfected water met the drinking water standards.

**TABLE 1:** Physicochemical and microbiological parameters measured at different treatment units for drinking water.

Samples	Temperature (°C)	pH	Turbidity (NTU)	DO (mg/L)	ATP (10^–8^ mol/L)	HPC (CFU/ml)	TOC (mg/L)	COD_Mn_ (mg/L)	TN (mg/L)
A	28.50 ± 0.26	7.63 ± 0.02	69.43 ± 2.29	6.31 ± 0.12	9.05 ± 0.02	3.11 × 10^4^	<0.5	1.49 ± 0.31	0.05 ± 0.01
B	27.10 ± 0	7.66 ± 0.02	69.47 ± 1.54	5.89 ± 0.22	4.11 ± 0.43	2.51 × 10^4^	<0.5	1.20 ± 0.12	0.04 ± 0.00
C	27.23 ± 0.06	7.88 ± 0.02	2.62 ± 0.12	6.99 ± 0.06	3.05 ± 0.01	2.50 × 10^3^	<0.5	0.70 ± 0.02	<0.02
E	27.53 ± 0.06	8.16 ± 0.01	2.62 ± 0.12	6.31 ± 0.09	1.84 ± 0.31	2.80 × 10^3^	<0.5	0.32 ± 0.01	<0.02
F	27.53 ± 0.06	8.07 ± 0.04	0.41 ± 0.05	6.89 ± 0.02	0.19 ± 0.06	0	<0.5	0.23 ± 0.02	<0.02
G	27.90 ± 0.04	7.93 ± 0.02	0.42 ± 0.02	6.46 ± 0.08	0.19 ± 0.06	10	<0.5	0.31 ± 0.03	<0.02

*A, raw water; B, flocculation tank effluent; C, sedimentation tank effluent; E, sand filter effluent; F, chlorine-disinfected water; G, tap water.*

### Bacterial Taxa Particularly Enriched in the Drinking Water Treatment System

**FIGURE 2:**
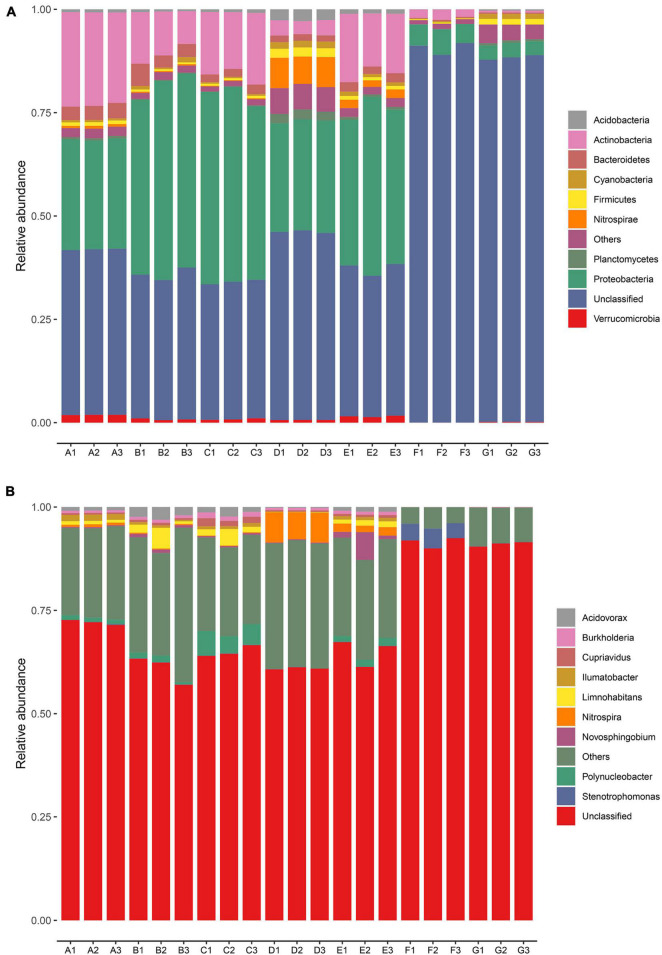
Relative abundance of bacterial community in the DWTS bulk water and sand biofilm at the phylum level **(A)** and genus level **(B)**. Sequences that could not be classified into any known phylum or genus are assigned as unclassified bacteria. The rare species with relative abundance <0.1% are assigned to others. A1–A3: raw water; B1–B3: flocculation tank effluent; C1–C3: sedimentation tank effluent; D1–D3: quartz sand; E1–E3: sand filter effluent; F1–F3: chlorine-disinfected water; G1–G3: tap water. Three samples were taken from each sampling site.

The dominant bacterial phyla and genera were identified based on the metagenomic data in every sampling site (Figure 2). A total of 95 bacterial phyla were identified. The relative abundances of the top nine bacteria at the phyla level in all samples are illustrated in Figure 2A. Proteobacteria and Actinobacteria were the dominant phyla in the DWTP, and they shifted largely during the water treatment process. After flocculation, the relative abundance of Proteobacteria was greatly improved and then remained at a certain ratio until chlorination, whereas the proportion of Actinobacteria decreased sharply after flocculation, followed by a slight increase after the settling process. However, the fate of the Actinobacteria was the same as the Proteobacteria; it decreased greatly after chlorination but maintained a certain ratio.

Previous studies have indicated that δ-Proteobacteria could be used as a biomarker to distinguish source water and tap water (Han et al., 2020). In other words, δ-Proteobacteria exhibit relatively low chlorine resistance, but α-Proteobacteria, β-Proteobacteria, γ-Proteobacteria, and Actinobacteria demonstrate strong resistance to the water treatment processes, including chlorination. Similarly, another author found that α-Proteobacteria and Actinobacteria were dominant in all the chlorine-disinfection samples (Li et al., 2020). Thus, in this study, we observed that the phyla of Proteobacteria and Actinobacteria were still predominant in the finished water. Moreover, the microbial community structure in the sand biofilm was significantly different from the water samples. The relative abundance of Nitrospirae, Planctomycetes, Chloroflexi, and Acidobacteria in the sand biofilm was higher than in the water samples. We also observed that the microbial community in the sand biofilm shaped the sand filter effluent. This finding is consistent with our previous study (Hou et al., 2018) and others (Oh et al., 2018; Zhou et al., 2021). Eustigmatophyceae, Firmicutes, and Planctomycetes in the finished water increased when they passed through the pipe network into the tap water, which might be involved in the regrowth and secondary contamination of bacteria in the pipe network (Douterelo et al., 2016). Some previous studies have demonstrated that the bacterial community is strongly correlated with the resistance profile (Jia et al., 2015; Zhang L. et al., 2018; Chen et al., 2019). In particular, Proteobacteria and Acidobacteria are important hosts of ARGs (Ma et al., 2019), and some studies have detected significant positive correlations between them and ARGs (Su et al., 2015; Xu et al., 2020). Thus, the changes in the relative abundance of Proteobacteria and Acidobacteria might explain the variations in the abundances of ARGs during the water treatment processes.

As evident in Figure 2B, at the genus level, the dominant genera were *Polynucleobacter*, *Acidovorax*, *Limnohabitans*, and *Nitrospira*, of which *Acidovorax* has been reported to be the host of ARGs in the DWTP (Jia et al., 2019). After chlorine disinfection, the relative abundance of *Stenotrophomonas* greatly increased and became the predominant bacteria. *Stenotrophomonas* has been reported to be typical chlorine-resistant bacteria (Luo et al., 2021), and it harbors many ARGs (Gil-Gil et al., 2020). Some studies reported that chlorine disinfection leads to an increase in the abundance of antibiotic-resistant bacteria (Khan et al., 2016), and chlorine disinfection affects the horizontal transfer of ARG through various pathways, including increasing the relative abundance of MGEs (Zhang et al., 2019). Moreover, some researchers have demonstrated that bacteria that survived chlorine disinfection might have higher levels of antibiotic resistance than other bacteria (Jia et al., 2015). Furthermore, the increased ARG abundance of chlorine-resistant bacteria might increase antibiotic resistance in pathogenic bacteria. Therefore, chlorine-resistant bacteria deserve attention.

### Antibiotic Resistome in the Drinking Water Treatment System

**FIGURE 3:**
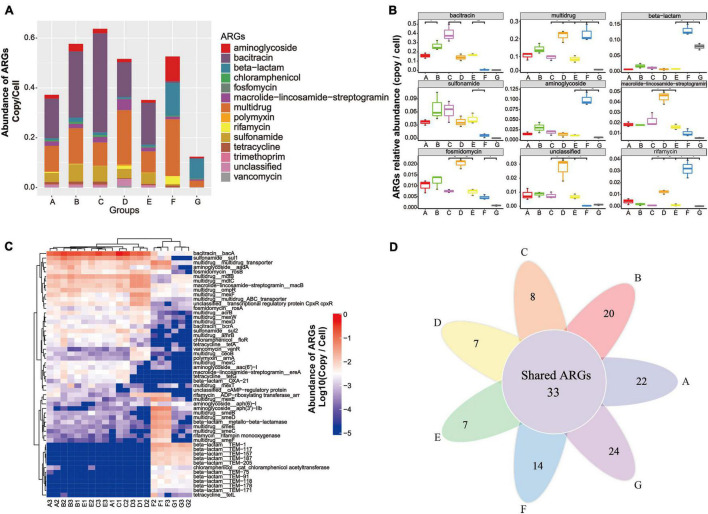
Occurrence and abundance of ARG types and ARG subtypes in the DWTS bulk water and sand biofilm. **(A)** Characterization of ARG types and abundance. **(B)** Boxplots showing the number of detected ARG types in the DWTS bulk water and sand biofilm. **(C)** Occurrence and fate of the top 20 ARG subtypes in the DWTS bulk water and sand biofilm. **(D)** Venn diagram of ARGs subtypes in the DWTS bulk water and sand biofilm. A1–A3: raw water; B1–B3: flocculation tank effluent; C1–C3: sedimentation tank effluent; D1–D3: quartz sand; E1–E3: sand filter effluent; F1–F3: chlorine-disinfected water; G1–G3: tap water. Three samples were taken from each sampling site.

Metagenomic data based on the structured SARG database were used to profile the antibiotic resistome in the DWTS. All the identified ARG-like sequences were classified into 13 ARG types (e.g., aminoglycoside and bacitracin resistance gene, as illustrated in Supplementary Material 1) according to the set script. As illustrated in Figure 3A, the abundance of 13 ARG types detected in all samples was normalized as the number of copies of ARGs per cell (copy/cell). Gene-encoded bacitracin (0.3 × 10^–2^ to 0.40 copies/cell, mean value = 0.23 ± 0.11 copies/cell), multidrug (0.02 to 0.23 copies/cell, mean value = 0.13 ± 0.05 copies/cell), and sulfonamide (0.2 × 10^–3^ to 0.7 × 10^–1^ copies/cell, mean value = 0.05 ± 0.02 copies/cell) ARGs were detected at the highest levels of abundance in the A–E samples. Previous studies have also reported that bacitracin, multidrug, and sulfonamide ARGs were predominant in drinking water samples (Ma et al., 2019; Zhang et al., 2020d).

However, aminoglycoside, beta-lactam, and rifamycin ARGs were notably enriched after chlorination. Moreover, in tap water, beta-lactam and multidrug ARGs retained a high abundance when they passed through the drinking water distribution system, which might threaten human health. From another perspective, the bacitracin ARG was mainly distributed in the B and C samples, which account for about 36 and 30%, respectively (Supplementary Figure 1 and Supplementary Material 2). The multidrug ARGs were mainly distributed in the B and F samples, which account for 20 and 26%, respectively (Supplementary Figure 1), while the sulfonamide ARGs were distributed in the A, B, C, and E samples. Similarly, as evident from Figure 3B, bacitracin and sulfonamide ARGs can be significantly removed by sand filters and chlorination. In other words, the DWTS effectively removed these ARGs, while the other ARGs cannot be eliminated very well. Some ARG types increased through the DWTS, such as multidrug, beta-lactam, aminoglycoside, and rifamycin (Figure 3B).

In total, 464 ARG subtypes were annotated (e.g., *bac*A, *sul*1 and multidrug_transporter, illustrated in Supplementary Material 1). Figure 3C illustrates the profile of the top 20 ARG subtypes during the drinking water treatment processes. Bacitracin_*bac*A (0.1 to 0.5 copies/cell) had the highest levels of abundance in the A–E samples, followed by sulfonamide_*sul*1 (0.2 × 10^–1^ to 0.9 × 10^–1^ copies/cell) and multidrug_*mdt*B (0.2 × 10^–1^ to 0.5 × 10^–1^ copies/cell). After chlorination, gene-encoded multidrug (*acr*B, *amr*B, and *ceo*B), bacitracin (*bcr*A), sulfonamide (*sul*1 and *sul*2), chloramphenicol (*flo*R), tetracycline (tetA and tetG), polymyxin (arnA), macrolide-lincosamide-streptogramin (*ere*A), and beta-lactam (OXA-21) decreased sharply. Previous studies have reported that *tetA* could be removed using free chlorine, and this is similar to our results (Amarasiri et al., 2020). However, some researchers have demonstrated that the abundance of *sul*1 and *sul*2 increased after chlorination (Liu et al., 2018; Zhu et al., 2021). Subsequently, the shift of these ARGs remains controversial when chlorine is used.

Additionally, multidrug (multidrug_transporter, *mex*E, *sme*B, *sme*C, *sme*D, *sme*E, and *sme*F), aminoglycoside [*aad*A, *aph* (6)-1, *aph* (3’)-IIb], beta-lactam (metallo-beta-lactamase, *TEM*-1, *TEM*-117, *TEM*-157, *TEM*-187, *TEM*-205, *TEM*-75, *TEM*-91, and *TEM*-118), and rifamycin (rifampin monooxygenase) were enriched. With respect to the phenomenon that some ARGs increased after chlorine disinfection, some researchers have pointed out that free chlorine could promote the transformation of ARGs through a series of cell responses, for example, raising levels of reactive oxygen species (ROS), ROS-mediated DNA damage, and bacterial membrane damage (Zhang et al., 2021). Our results indicated that chlorination reduced the diversity of the ARG types. Additionally, the Venn diagrams for the different drinking water treatment processes display the number of unique and shared ARG subtypes at each sampling site (Figure 3D). The shared 33 ARG subtypes, which constituted 10% of the total ARG subtypes, were universally present from the raw water to tap water (Supplementary Material 3). The shared ARG subtypes were distributed in aminoglycoside (*n* = 1), aminoglycoside (*n* = 2), bacitracin (*n* = 2), beta-lactam (*n* = 2), fosmidomycin (*n* = 2), macrolide-lincosamide-streptogramin (*n* = 2), multidrug (*n* = 18), rifamycin (*n* = 1), tetracycline (*n* = 1), vancomycin (*n* = 1), and unclassified (*n* = 1), respectively. Thus, the largest variety of shared ARG subtypes belonged to the multidrug ARGs. These shared ARG subtypes persistent in the DWTS will lead to continuous pollution. It is also worth noting that the unique ARG subtypes in process F (*n* = 14) that escaped from chlorine disinfection were assigned to beta-lactam (*n* = 11), followed by macrolide-lincosamide-streptogramin (*n* = 2) and chloramphenicol (*n* = 1), respectively. Furthermore, the unique ARG subtypes in process G (*n* = 24) were assigned to aminoglycoside (*n* = 1), beta-lactam (*n* = 18), bleomycin (*n* = 1), macrolide-lincosamide-streptogramin (*n* = 2), multidrug (*n* = 1), and vancomycin (*n* = 1), respectively. Therefore, it is evident that the unique ARG subtypes in tap water rebounded when passed through the distribution system, which requires further attention. Shared ARG subtypes or unique ARG subtypes in the processes F and G pose a risk to human health when people drink the water. Additionally, these ARG subtypes might be transmitted to human living areas; for example, when people use water to wash food, such as fruit and vegetables, the ARGs will contaminate the fruit and vegetable surfaces and affect health through the food chain. Recently, scholars have discovered that ARGs in vegetables or fruit pose risks to ecological environment health (Sun et al., 2021). Furthermore, when people use the water to take a bath, the ARG subtypes might also remain on the skin (Nielsen and Jiang, 2019).

### Antibiotic Resistance Genes Host Pattern in the Drinking Water Treatment System

**FIGURE 4:**
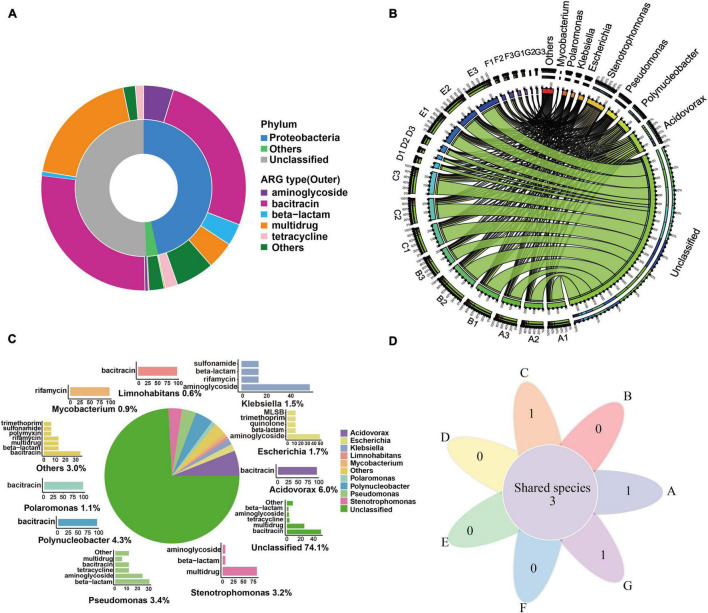
ARG hosts analysis in the DWTS. **(A)** Annotation of ARG-carrying contigs at the phylum level. **(B)** The circle diagram shows the taxonomy and proportion of putative ARG hosts in DWTS. **(C)** The taxonomy of ARG-carrying contigs (in the genus level) and the percentages of ARG types these contigs carried. a: Pie chart shows the taxonomy and percentage of ARG-carrying contigs. For example, *Acidovorax* (6.0%) denotes that 6.0% of ARG-carrying contigs were annotated as *Acidovorax*. b: Bar chart shows the percentages of ARGs types that were carried by the annotated ARG-carrying contigs. The percentage of these annotated ARG-carrying contigs was set as 100%. For example, 100% of the ARG-carrying contigs originating from *Acidovorax* spp. carried bacitracin resistance genes. **(D)** Venn diagrams showing the number of shared species that carried ARG subtypes in DWTS bulk water and sand biofilm. A1–A3: raw water; B1–B3: flocculation tank effluent; C1–C3: sedimentation tank effluent; D1–D3:quartz sand; E1–E3: sand filter effluent; F1–F3: chlorine-disinfected water; G1–G3: tap water. Three samples were taken from each sampling site.

As evident from Figure 4A, most of the ACCs were annotated as fragments of *Proteobacteria* (46.6%). Additionally, these contig-harbored ARGs were mainly resistant to bacitracin (26.08%), aminoglycoside (4.74%), multidrug (4.31%), and beta-lactam (3.45%). Similarly, the Proteobacteria phylum was identified as the most abundant bacterial host of ARGs (25.3–94.6%) in DWTPs (Zhao et al., 2022). Proteobacteria phylum was also regarded as a major pool of ARGs in sewage sludge (Xu et al., 2018), industrial mariculture systems (Wang et al., 2018), and the human gut (Hu et al., 2016). This is supported by the findings of our research. As illustrated in Figure 4B, before chlorine disinfection (processes A–E), the main putative ARG hosts were *Acidovorax* and *Polynucleobacter*, which were also the dominant bacteria in the raw water (Figure 2B and Supplementary Table 2). In the flocculation tank effluent, we found that *Acidovorax* and *Stenotrophomonas* were the main putative ARG hosts, while, in the sedimentation effluent, the main putative ARG carriers were *Acidovorax*, *Polynucleobacter*, and *Stenotrophomonas*. The main ARG carrier in the quartz sand sample was *Pseudomonas*, whereas *Nitrospira*, the predominant bacteria in the quartz sand biofilm, was not the dominant ARG host. Therefore, sand filters might be low risk for the transmission of ARGs. In the sand filter effluent, the main ARG carrier was *Limnohabitans*. Therefore, in general, we can see that *Acidovorax* and *Stenotrophomonas* were the most abundant ARG carriers of bacteria before chlorine disinfection.

However, after chlorine disinfection (processes F and G), the main ARG carriers were *Stenotrophomonas* and *Escherichia*. Interestingly, after the chlorine disinfection, the relative abundance of *Stenotrophomonas* also greatly increased and became the predominant bacteria (Figure 2B). *Stenotrophomonas* has been reported to be a highly chlorine-resistant Gram-negative bacterium that can regrow post disinfection (Shekhawat et al., 2020, 2021), and it is regarded as a global emerging Gram-negative multiple drug-resistant organism (Brooke, 2012). Similarly, *Escherichia* was widely reported to be an indicator of drug-resistant genes (Zhang et al., 2020e) and easily escaped the oxidation by disinfectants (Mounaouer and Abdennaceur, 2016). We assumed that these bacteria might regrow in the drinking water distribution system, or there was secondary pollution in the pipe network. Therefore, these bacteria are of high concern for public health safety in drinking water environments.

From all the water samples, as illustrated in Figure 4C, the dominant putative ARG hosts include *Acidovorax* (6.0%), *Polynucleobacter* (4.3%), *Pseudomonas* (3.4%), *Escherichia* (1.7%), *Klebsiella* (1.5%), *Polaromonas* (1.1%), *Mycobacterium* (0.9%), and *Limnohabitans* (0.6%). *Acidovorax* was the most abundant bacterium that carried bacitracin ARGs as *Polynucleobacter*, *Polaromonas*, and *Limnohabitans*. A previous study also reported that *Acidovorax* was the main carrier of bacitracin_*bac*A (Asante and Sekyere, 2019), and *Polynucleobacter* was an abundant ARG host in drinking water sources and frequently carried bacitracin ARGs (Bai et al., 2019). It was reported that the putative ARG host distribution pattern might be most likely responsible for the overall ARG profile (Bai et al., 2019). Therefore, these bacteria were most likely to drive the shift of bacitracin ARGs. *Mycobacterium* drove the shift of rifamycin ARGs, and *Pseudomonas*, *Escherichia*, and *Klebsiella* carried the largest variety of ARGs. According to the further analysis of ARG-carrying contigs (ACCs), we found that 49 ACCs were annotated as pathogens (Supplementary Material 4). They were mainly classified as *Stenotrophomonas maltophilia* (*n* = 15), *P. aeruginosa* (*n* = 12), *E. coli* (*n* = 7), *Klebsiella pneumoniae* (*n* = 6), *Acinetobacter baumannii* (*n* = 3), *Rhodococcus erythropolis* (*n* = 2), *Pseudomonas putida* (*n* = 2), *Mycobacterium smegmatis* (*n* = 1), and *Enterobacter aerogenes* (*n* = 1). Thus, we can see that the most common pathogens were *S. maltophilia* and *P. aeruginosa* in the DWTS. Moreover, *Escherichia* spp. mainly carried tetracycline and sulfonamide ARGs (Figure 4C), while *Stenotrophomonas* spp. mainly carried aminoglycoside, beta-lactam, and multidrug ARGs (Figure 4C). Additionally, pathogens were distributed in processes A (*n* = 41), B (*n* = 40), C (*n* = 40), D (*n* = 12), E (*n* = 41), F (*n* = 26), and G (*n* = 20). In particular, *S. maltophilia* was persistent in every process (Figure 4D and Supplementary Material 5), and the sustained survival of these bacteria in the DWTS could lead to the horizontal propagation of ARGs and even increase risks to human health.

### Distribution of Antibiotic Resistance Genes in Mobile Elements

**FIGURE 5:**
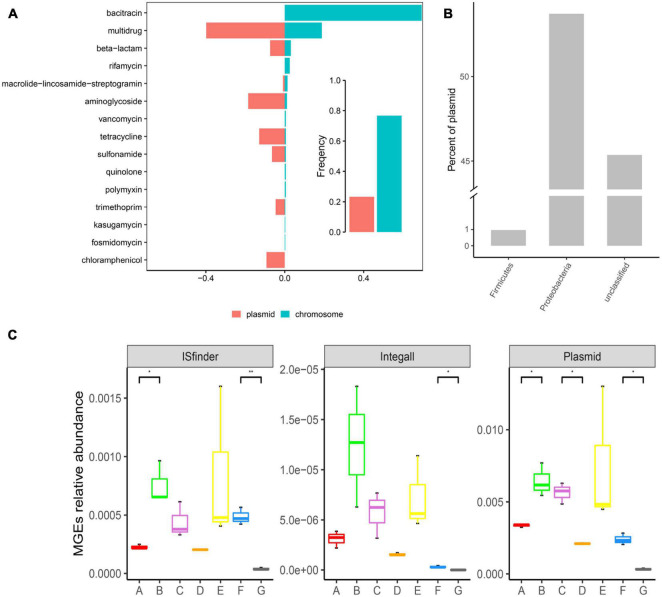
Distribution of ARGs in mobile genetic elements (MGEs) and distribution of MGEs in the DWTS. **(A)** Occurrence and abundance of ARG types in MGEs. Bar plot showing the frequency of ARGs in bacterial chromosomes (blue) or plasmids (red) summarized by antibiotic class. **(B)** Distribution of plasmids in bacteria (at the phylum level). **(C)** Distribution of MGEs in the DWTS. A: raw water; B: grid reaction tank effluent; C: settling pond effluent; D: quartz sand; E: sand filter effluent; F: chlorine-disinfected water; G: tap water.

As illustrated in Figure 5A, ARGs present in the DWTS were more prevalent in bacterial chromosomes than in plasmids. Specifically, ARGs for bacitracin, rifamycin, vancomycin, quinolone, polymyxin, kasugamycin, and fosmidomycin were just prevalent in chromosomes. These ARGs might not contribute to horizontal gene transfer. While ARGs for multidrug, beta-lactam, aminoglycoside, tetracycline, sulfonamide, trimethoprim, and chloramphenicol were prevalent mainly in plasmids. Significantly, chloramphenicol was only presented in plasmids. Therefore, we must focus on these kinds of ARGs with a higher risk of transmission. Additionally, the phylum of Proteobacteria harbored the highest abundance of plasmids (Figure 5B), followed by Firmicutes. Meanwhile, the abundance of plasmids was higher than that of insert sequence (IS) and integron (Figure 5C). The most abundant MGE plasmids appeared in the sand filter effluent, followed by the flocculation tank effluent.

However, in the quartz sand biofilm, the abundance of MGE plasmids was the lowest, and the distribution trend of MGE ISfinder in drinking water treatment processes was similar to the MGE plasmids. Interestingly, ARG abundance in the quartz sand biofilm was also the lowest (Supplementary Figure 2). At the same time, the Shannon index indicated that microbial diversity was the most abundant in the quartz sand biofilm. These results demonstrate that the probability of horizontal transmission of ARGs was the least in the quartz sand filters and the highest in the flocculation tank effluent. Integron class1 is a major player in disseminating antibiotic resistance genes across pathogens and commensals. Therefore, in this study, we also looked for integron class1 and found a higher prevalence in the DWTS; the abundance of integron class1 in the flocculation tank effluent water was the highest than the other samples (Supplementary Figure 3). Additionally, it decreased after chlorine disinfection and significantly decreased in the process of drinking water transmission, as well as MGE plasmids and MGE ISfinder. Plasmids and integrons are prone to recombination (Mazel, 2006); thus, chlorine disinfection and residual chlorine in the distribution system could prevent the dissemination of ARGs.

### Co-occurrence Patterns Between Antibiotic Resistance Gene Subtypes

**FIGURE 6:**
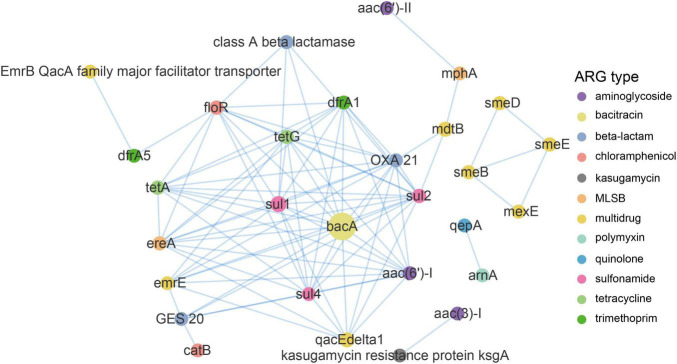
Network analysis demonstrating the co-occurrence patterns of ARG subtypes in the DWTS. The nodes were colored according to network modularity, and the node size is proportional to the number of connections (degree). An edge is a strong (ρ > 0.8) and significant (*p*-value <0.01) connection between nodes.

A network analysis based on the metagenomic approach was used to discern the co-occurrence patterns between ARG subtypes and demonstrated a good advantage (Ju et al., 2016; Zhang et al., 2020a). As illustrated in Figure 6 and Supplementary Table 3, a total of 90 pairs of ARG subtypes under a cutoff of *p* < 0.01 and ρ > 0.8 were discovered from 29 ARG subtypes assigned to 12 ARG types. ARG subtypes assigned to the same ARG type (i.e., intra-types) tend to be co-occurring. Overall, ten out of the 90 pairs were intra-type co-occurrence correlations. For instance, *emr*E and *qacEdelta*1 for multidrug resistance correlated (*r* = 0.85), as well as the *sul*1 and *sul*2 (*r* = 0.93) for sulfonamide resistance and *tet*A and *tet*G (*r* = 0.88; Supplementary Table 3) for tetracycline resistance. Co-occurrence patterns were also observed between different ARGs subtypes (i.e., inter-types). For example, aac (3)-I for aminoglycoside resistance strongly correlated with *ksg*A (*r* = 1) for kasugamycin resistance, *bac*A for bacitracin resistance correlated with *sul*1 for sulfonamide resistance (*r* = 0.96), *flo*R for chloramphenicol resistance correlated with *tet*G for tetracycline resistance (0.95), and *qacEdelta*1 for multidrug resistance correlated with *sul*1 for sulfonamide resistance (0.93). Recent research also addressed the significant positive correlations among these ARG types and subtypes (Zhang S. et al., 2018; Zhang et al., 2020a).

### Co-occurrence Patterns Among Antibiotic Resistance Genes Subtypes, Mobile Genetic Elements, and Bacterial Taxa

A network analysis was performed to explore the co-occurrence patterns among ARG subtypes, MGEs, and bacterial genera. Co-occurrence patterns between ARG subtypes and bacterial genera could be a good way to track the potential hosts of ARGs in their environment. We hypothesized that the ARGs and bacterial taxa had a strong and significant positive correlation under a cutoff of *p* < 0.01 and ρ > 0.8. As illustrated in Supplementary Figure 4 and Supplementary Material 6, the co-occurrence results indicated that 42 genera might be potential hosts for 27 ARG subtypes. Among the 42 bacterial genera, *Acidovorax*, *Curvibacter*, *Variovorax*, *Comamonas*, and *Hydrogenophaga* carried more diverse ARG subtypes (14, 14, 13, 12, and 12, respectively) than other genera. For example, *Acidovorax* was identified as the possible host of the multidrug-resistance gene (*emr*E and qacEdeltal), sulfonamide-resistance gene (e.g., *sul*1 and *sul*2), tetracycline-resistance gene (*tet*A and *tet*G), trimethoprim-resistance gene (*dfrA*1), beta-lactam-resistance gene (OXA-21 and class-A-beta-lactamase), chloramphenicol-resistance gene (*floR*), bacitracin-resistance gene (*bac*A), macrolide-lincosamide-streptogramin-resistance gene (*ere*A), and aminoglycoside-resistance gene [*aac*(6_)-I] (Supplementary Material 6). Likewise, *Curvibacter* was the possible host of the bacitracin-resistance gene (*bac*A), beta-lactam-resistance gene (OXA-21 and class-A-beta-lactamase), multidrug-resistance gene (*emr*E and *qacEdelta*l), sulfonamide-resistance genes (e.g., *sul*1 and *sul*2), tetracycline-resistance gene (*tet*A and *tet*G), and trimethoprim-resistance gene (*dfr*A1).

Additionally, a total of 57 pairs with *p* < 0.01 and ρ > 0.8 were observed between the MGEs and bacterial genera. For instance, *intI*1 had a significant and positive correlation with ten bacterial genera (Supplementary Material 6). IS21 correlated positively and significantly with 15 bacterial genera, and IS3 had a strong positive collection with 10 bacterial genera. Furthermore, a total of 31 pairs with *p* < 0.01 and ρ > 0.8 were observed between the MGE and ARG subtypes. *IntI*1 had a significantly positive correlation with eight ARG subtypes, and IS21 had a significantly positive correlation with nine ARG subtypes. These results implied the important role of *IntI*1 and *IS*21 in antibiotic resistance dissemination. It has been reported that MGE-associated ARGs tend to persist in the treatment at DWTPs and might pose a higher risk of resistance dispersal (Zhao et al., 2022). Similarly, recent literature has also reported the increase in the proportion of MGE-associated ARGs in wastewater treatment plants (Che et al., 2019).

### Bins

**FIGURE 7:**
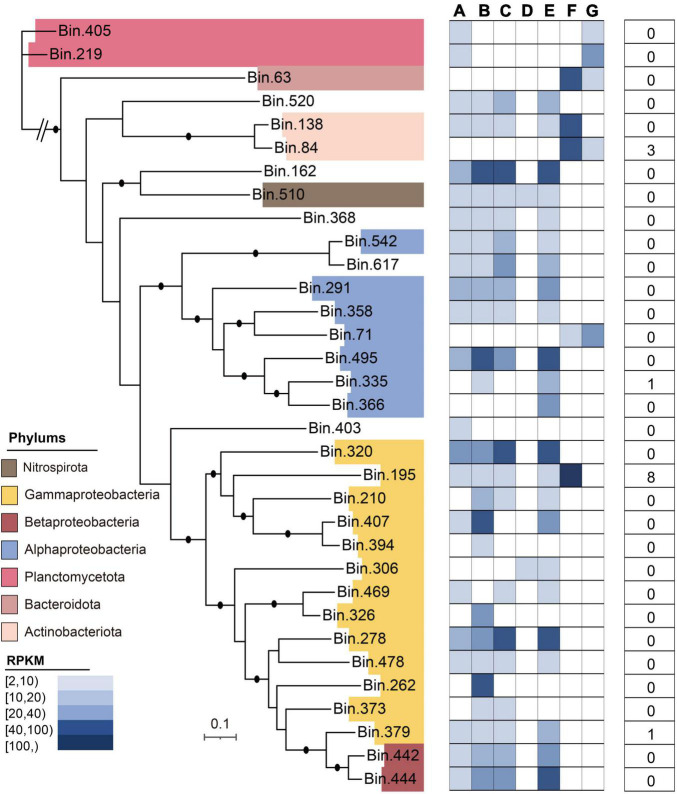
Taxonomic assignment of 33 MAGs reconstructed from the DWTS. The phylogenomic tree was constructed using 31 commonly conserved protein-coding genes. The average relative abundances [in reads per kilobase per million (RPKM)] of all MAGs in drinking water production processes are shown as a heat map, followed by data indicating the presence of the total ARG subtype numbers in MAGs.

In this study, after metagenomic binning, 33 high-quality metagenome-assembled genomes (MAGs) were obtained, which mainly included Gammaproteobacteria (*n* = 13), Alphaproteobacteria (*n* = 5), Betaproteobacteria (*n* = 2), Planctomycetota (*n* = 2), Bacteroidota (*n* = 1), Actinobacteriota (*n* = 2), and Nitrospirota (*n* = 1) (Figure 7). The general characteristics of these 33 MAGs are illustrated in Supplementary Table 4. Of the 33 MAGs, the phylum Proteobacteria (*n* = 20) dominated the microbial community in the DWTS, followed by Planctomycetota (*n* = 2) and Actinobacteriota (*n* = 2). The MAG identified as *S. maltophilia*-like (bin.195) harbored the greatest number of ARG subtypes (*n* = 8), including multidrug (*n* = 6; *sme*D, *sem*E, multidrug_transporter, *mex*E, *sem*B, and *sme*C), beta-lactam (*n* = 1; metallo-beta-lactamase), and aminoglycoside [*n* = 1; aph(3’)-IIb] (Supplementary Table 5). Meanwhile, *Burkholderiaceae*-like (bin.379) also contained bacitracin (*n* = 1; *bac*A). Additionally, the MAG (bin.84) identified as *R. erythropolis*-like contained aminoglycoside [*n* = 2; ant (3”)-Ih-aac (6’)-Iid and ant (2”)-I] and rifamycin (*n* = 1; rifampin monooxygenase). Interestingly, based on our genome data of strain F2 identified to the genus of *Stenotrophomonas* sp., which also harbored *sme*D, *sem*E, multidrug_transporter, *sem*B, and *sme*C (Supplementary Material 7 and Supplementary Figure 5), these results can be verified against each other.

### Isolation and Identification of Bacteria From the Drinking Water Treatment System

A total of 93 isolates were recovered from the DWTS using traditional culture technology (Supplementary Tables 6, 7). The isolates belong to 30 genera based on a 16S rRNA gene sequencing analysis, and they were found to belong to five phyla: Proteobacteria, Firmicutes, Actinobacteria, Deinococcus-Thermus, and Bacterioidetes. Proteobacteria (42%) and Firmicutes (38%) were the dominant phyla. As illustrated in Supplementary Table 7, at the genus level, *Bacillus* was the most abundant genus (20%), followed by *Pseudomonas* (11%) and *Acidovorax* (9%), respectively. Moreover, 47 isolates within 20 genera were recovered from the sand filter, representing the maximum microbial diversity in all the sampling sites. Bacteria isolated from this process accounted for 51% of all the isolates, meaning that bacteria in the sand filter were the most abundant. These results were consistent with the microbial diversity analysis based on the metagenomic data in this study.

It is worth noting that some pathogens and opportunistic pathogens such as *P. aeruginosa*, *Bacillus cereus*, *Staphylococcus hominis*, *E. coli*, and *Aeromonas enteropelogenes* were detected in water treatment processes. Fortunately, only one bacterium was detected after chlorination, and it was not a pathogen or opportunistic pathogen, which indicated that chlorination effectively removed high-risk bacteria in the DWST. However, it does not demonstrate that the tap water was completely microbial safe. In our study, five isolates were obtained from the tap water, indicating secondary pollution in the drinking water pipe network. Previous literature has reported that disinfectant could induce culturable bacteria and various pathogenic bacteria into a viable but non-culturable (VBNC) state (Lin et al., 2017) in the DWTS, which would result in these bacteria not being detected using traditional culture methods. Therefore, a comprehensive microbiological safety assessment of the drinking water could combine molecular methods with culture-based methods.

### Antimicrobial Susceptibility Testing

The antimicrobial susceptibility testing results (Table 2) demonstrated that selected isolates had high resistance to FOX (56%), 48% of the selected isolates were resistant to P, and 28% were resistant to AMC. However, the resistance to TOB, DA, and E was lower (4, 8, and 8%, respectively). In the Proteobacteria, 88% of the isolates were resistant to AMC and 63% were resistant to AMP. However, the incidence of resistance to TE, TOB, CIP, CAZ, and MEM was very low. In the Firmicutes, 71% of the isolates were resistant to FOX, 59% to P, and only 12% to TE, DA, and E. Bacteria were regarded as multidrug resistant (MDR) if they were resistant to three or more groups of antibiotics. In this study, 28% of the selected isolates belong to MDR, and the occurrence of MDR phenotypes was mainly related to the bacterial species. The MARI was applied to verify the origin of the antibiotic resistance in the isolates. The MARI value of the selected isolates recovered from the DWTS was 0.09–0.50. Compared to other environments, the MARI value of the isolates that recovered from the DWTS was lower. For example, the MARI value of *Salmonella enterica* that recovered from broiler chickens and retail shops was 0.2–0.6 (Elkenany et al., 2019), and *E. coli* that isolated from clinical specimens, including eye and blood cells, had higher MARI values of 0.82 and 0.74, respectively (Masoud et al., 2021). Additionally, according to the MARI value, the B3, B11, D38, D20, and F2 isolates might be from a source where antibiotics were used frequently.

**TABLE 2:** Antibiotic resistance profiles of the selected species isolated from the DWTS.

Number	Isolate species	Phylum level	Isolate number	Resistotype	MARI	MDR
1	*Pseudomonas aeruginosa*	*Proteobacteria*	B3	CRO-P-CTX-AMP-SXT-K-AMC-TE	(0.44) 8/18	1
2	*P. aeruginosa*	*Proteobacteria*	B11	P-CTX-AMP-AMC-CN	(0.28) 5/18	1
3	*P. alcaligenes*	*Proteobacteria*	D15	TOB	(0.13) 1/18	0
4	*P. alcaligenes*	*Proteobacteria*	D38	P-AMP-SXT-AMC-CAZ	(0.28) 5/18	1
5	*P. otitidis*	*Proteobacteria*	D44	AMC	(0.13) 1/18	0
6	*B. cereus*	*Firmicutes*	D5	P-FOX	(0.18) 2/11	0
7	*B. cereus*	*Firmicutes*	D20	P-FOX-TE	(0.27) 3/11	1
8	*B. cereus*	*Firmicutes*	D33	P-FOX	(0.18) 2/11	0
9	*B. cereus*	*Firmicutes*	D46	FOX	(0.09) 1/11	0
10	*B. altitudinis*	*Firmicutes*	D8	DA-P	(0.18) 2/11	0
11	*B. subtilis*	*Firmicutes*	B9	FOX	(0.09) 1/11	0
12	*B. toyonensis*	*Firmicutes*	C3	P-FOX	(0.18) 2/11	0
13	*B. aerophilus*	*Firmicutes*	D13	DA	(0.09) 1/11	0
14	*B. stratosphericus*	*Firmicutes*	D14	FOX	(0.09) 1/11	0
15	*B. pumilus*	*Firmicutes*	D16	FOX-TE	(0.18) 2/11	0
16	*B. wiedmannii*	*Firmicutes*	D21	P-FOX	(0.18) 2/11	0
17	*B. thuringiensis*	*Firmicutes*	D26	P-FOX	(0.18) 2/11	0
18	*Staphylococcus warneri*	*Firmicutes*	C2	P-E	(0.18) 2/11	0
19	*S. haemolyticus*	*Firmicutes*	D41	FOX	(0.09) 1/11	0
20	*S. haemolyticus*	*Firmicutes*	E2	P-E	(0.18) 2/11	0
21	*S. warneri*	*Firmicutes*	G1	P	(0.09) 1/11	0
22	*S. hominis*	*Firmicutes*	G3	FOX	(0.09) 1/11	0
23	*Enterobacter tabaci*	*Proteobacteria*	B17	FOX-AMC-CN	(0.15) 3/20	1
24	*Stenotrophomonas maltophilia*	*Proteobacteria*	F2	NOR-CRO-CIP-CTX-AMP-MEM-FOX-K-AMC	(0.50) 9/18	1
25	*Klebsiella pneumoniae*	*Proteobacteria*	B19	AMP-AMC-CN	(0.15) 3/20	1

## Conclusion

In this study, metagenomic approaches were applied to comprehensively profile the pattern of the microbial community structure, ARGs, and MGEs in the DWTS. The results indicated that Proteobacteria, Acidobacteria, Bacteroidetes, and Firmicutes were the dominant phyla in the DWTS and shifted largely during the water treatment process. Additionally, gene-encoded bacitracin (0.3 × 10^–2^ to 0.40 copies/cell), multidrug (0.02–0.23 copies/cell), and sulfonamide (0.2 × 10^–3^ to 0.7 × 10^–1^ copies/cell) were detected at the highest levels of abundance before chlorination, while the aminoglycoside, beta-lactam, and rifamycin ARGs were notably enriched after chlorination. Among the drinking water treatment processes, chlorination profoundly influenced the ARG composition. A total of 317 ARG subtypes were annotated, and bacitracin_*bac*A (0.1–0.5 copies/cell) was generally the most prevalent subtype in all samples. The taxonomy results demonstrated that the dominant putative ARG hosts included *Acidovorax* (6.0%), *Polynucleobacter* (4.3%), *Pseudomonas* (3.4%), *Escherichia* (1.7%), and *Klebsiella* (1.5%). A further analysis indicated that 49 ACCs were annotated as pathogens. The most common pathogens were *S. maltophilia* and *P. aeruginosa* in the DWTS. Additionally, the network analyses suggested that 42 genera were deduced as the potential hosts of the 27 ARG subtypes. The metagenomic binning results demonstrated that 33 high-quality MAGs were recovered from the DWTS and bin.195 harbored the greatest number of ARG subtypes (*n* = 8), including multidrug (*n* = 6; *sme*D, *sem*E, multidrug_transporter, *mex*E, *sem*B, and *sme*C), beta-lactam (*n* = 1; metallo-beta-lactamase), and aminoglycoside [*n* = 1; aph(3’)-IIb]. In total, ninety-three isolates were obtained from the DWTS, and 28% of the selected isolates were MDR. Additionally, some pathogens and opportunistic pathogens such as *P. aeruginosa*, *B. cereus*, *S. hominis*, *E. coli*, and *A. enteropelogenes* were isolated from the DWTS. The occurrence of these pathogenic bacteria demonstrates that higher requirements for disinfection technology are necessary and might threaten the safety of drinking water. Our findings provide comprehensive insights into the occurrence and dissemination of ARGs and ARB in the DWTS.

## Data Availability Statement

Raw data have been submitted to the NCBI Sequence Read Archive (SRA) under accession numbers SRR17063149-17063169.

## Author Contributions

QW and QG conceived and designed the experiments. QG, TL, and MS performed the experiments. QG, SW, SZ, YZ, RP, and XW analyzed the data. QW, YD, JW, ZL, LC, WC, XL, and JZ contributed reagents, materials, and analysis tools. QG wrote the manuscript. MC, LX, and QG reviewed the manuscript. All authors contributed to the article and approved the submitted version.

## Conflict of Interest

The authors declare that the research was conducted in the absence of any commercial or financial relationships that could be construed as a potential conflict of interest.

## Publisher’s Note

All claims expressed in this article are solely those of the authors and do not necessarily represent those of their affiliated organizations, or those of the publisher, the editors and the reviewers. Any product that may be evaluated in this article, or claim that may be made by its manufacturer, is not guaranteed or endorsed by the publisher.

## Footnotes

1
https://www.ncbi.nlm.nih.gov/

2
https://github.com/hyattpd/Prodigal

3
https://www.nature.com/articles/s41467-018-04964-5

4
http://sourceforge.net/projects/bbmap/

## References

[B1] Amarasiri M. Sano D. Suzuki S. (2020). Understanding human health risks caused by antibiotic resistant bacteria (ARB) and antibiotic resistance genes (ARG) in water environments: current knowledge and questions to be answered. *Crit. Rev. Environ. Sci. Technol.* 50 2016–2059. 10.1080/10643389.2019.1692611

[B2] Asante J. Sekyere J. O. (2019). Understanding antimicrobial discovery and resistance from a metagenomic and metatranscriptomic perspective: advances and applications. *Environ. Microbiol. Rep.* 11 62–86. 10.1111/1758-2229.12735 30637962

[B3] Bai Y. Ruan X. H. Li R. F. Zhang Y. P. Wang Z. Z. (2021). Metagenomics-based antibiotic resistance genes diversity and prevalence risk revealed by pathogenic bacterial host in Taihu Lake, China. *Environ. Geochem. Health* 10.1007/s10653-021-01021-x 34292452

[B4] Bai Y. Ruan X. Xie X. Yan Z. (2019). Antibiotic resistome profile based on metagenomics in raw surface drinking water source and the influence of environmental factor: a case study in Huaihe River Basin, China. *Environ. Pollut.* 248 438–447. 10.1016/j.envpol.2019.02.057 30826606

[B5] Bergeron S. Boopathy R. Nathaniel R. Corbin A. Lafleur G. (2015). Presence of antibiotic resistant bacteria and antibiotic resistance genes in raw source water and treated drinking water. *Int. Biodeterior. Biodegrad.* 102 370–374. 10.1016/j.ibiod.2015.04.017

[B6] Bergeron S. Raj B. Nathaniel R. Corbin A. Lafleur G. (2017). Presence of of antibiotic resistance genes in raw source water of a drinking water treatment plant in a rural community of USA. *Int. Biodeterior. Biodegrad.* 124 3–9. 10.1016/j.ibiod.2017.05.024

[B7] Brooke J. S. (2012). *Stenotrophomonas maltophilia*: an emerging global opportunistic pathogen. *Clin. Microbiol. Rev.* 25 2–41. 10.1128/CMR.00019-11 22232370 PMC3255966

[B8] Camacho C. Coulouris G. Avagyan V. Ma N. Papadopoulos J. Bealer K. (2009). BLAST plus : architecture and applications. *BMC Bioinform.* 10:421. 10.1186/1471-2105-10-421 20003500 PMC2803857

[B9] Che Y. Xia Y. Liu L. Li A. D. Yang Y. Zhang T. (2019). Mobile antibiotic resistome in wastewater treatment plants revealed by Nanopore metagenomic sequencing. *Microbiome* 7:44. 10.1186/s40168-019-0663-0 30898140 PMC6429696

[B10] Chen Y.-R. Guo X.-P. Feng J.-N. Lu D.-P. Niu Z.-S. Tou F.-Y. (2019). Impact of ZnO nanoparticles on the antibiotic resistance genes (ARGs) in estuarine water: ARG variations and their association with the microbial community. *Environ. Sci. Nano* 6 2405–2419. 10.1039/C9EN00338J

[B11] CLSI (2020). *“Performance Standards for Antimicrobial Susceptibility Testing; Twenty-Fifth Informational Supplement. Approved Standard-M02-A12”.* Malvern, PA: The Clinical and Laboratory Standards Institute.

[B12] Douterelo I. Husband S. Loza V. Boxall J. (2016). Dynamics of biofilm regrowth in drinking water distribution systems. *Appl. Environ. Microbiol.* 82 4155–4168. 10.1128/AEM.00109-16 27208119 PMC4959196

[B13] Elkenany R. Elsayed M. M. Zakaria A. I. El-Sayed S. A. Rizk M. A. (2019). Antimicrobial resistance profiles and virulence genotyping of *Salmonella enterica* serovars recovered from broiler chickens and chicken carcasses in Egypt. *BMC Vet. Res.* 15:1. 10.1186/s12917-019-1867-z 31029108 PMC6486964

[B14] Fang H. Wang H. Cai L. Yu Y. (2015). Prevalence of antibiotic resistance genes and bacterial pathogens in long-term manured greenhouse soils as revealed by metagenomic survey. *Environ. Sci. Technol.* 49 1095–1104. 10.1021/es504157v 25514174

[B15] Gil-Gil T. Martinez J. L. Blanco P. (2020). Mechanisms of antimicrobial resistance in *Stenotrophomonas* maltophilia: a review of current knowledge. *Exp. Rev. Anti Infect. Therapy* 18 335–347. 10.1080/14787210.2020.1730178 32052662

[B16] Gu Q. Wu Q. Zhang J. Guo W. Ding Y. Wang J. (2018). Isolation and transcriptome analysis of phenol-degrading bacterium from carbon-sand filters in a full-scale drinking water treatment plant. *Front. Microbiol.* 9:2162. 10.3389/fmicb.2018.02162 30298058 PMC6160575

[B17] Han Z. An W. Yang M. Zhang Y. (2020). Assessing the impact of source water on tap water bacterial communities in 46 drinking water supply systems in China. *Water Res.* 172:115469. 10.1016/j.watres.2020.115469 31954932

[B18] Hou L. Zhou Q. Wu Q. Gu Q. Sun M. Zhang J. (2018). Spatiotemporal changes in bacterial community and microbial activity in a full-scale drinking water treatment plant. *Sci. Total Environ.* 625 449–459. 10.1016/j.scitotenv.2017.12.301 29291559

[B19] Hu Y. Yang X. Li J. Lv N. Liu F. Wu J. (2016). The bacterial mobile resistome transfer network connecting the animal and human microbiomes. *Appl. Environ. Microbiol.* 82 6672–6681. 10.1128/AEM.01802-16 27613679 PMC5086561

[B20] Ishii S. I. Suzuki S. Norden-Krichmar T. M. Tenney A. Chain P. S. G. Scholz M. B. (2013). A novel metatranscriptomic approach to identify gene expression dynamics during extracellular electron transfer. *Nat. Commun.* 4:1601. 10.1038/ncomms2615 23511466

[B21] Jia S. Shi P. Hu Q. Li B. Zhang T. Zhang X.-X. (2015). Bacterial community shift drives antibiotic resistance promotion during drinking water chlorination. *Environ. Sci. Technol.* 49 12271–12279. 10.1021/acs.est.5b03521 26397118

[B22] Jia S. Wu J. Ye L. Zhao F. Li T. Zhang X.-X. (2019). Metagenomic assembly provides a deep insight into the antibiotic resistome alteration induced by drinking water chlorination and its correlations with bacterial host changes. *J. Haz. Mater.* 379:120841. 10.1016/j.jhazmat.2019.120841 31279312

[B23] Jiang H. Zhou R. Zhang M. Cheng Z. Li J. Zhang G. (2018). Exploring the differences of antibiotic resistance genes profiles between river surface water and sediments using metagenomic approach. *Ecotoxicol. Environ. Saf.* 161 64–69. 10.1016/j.ecoenv.2018.05.044 29859409

[B24] Ju F. Li B. Ma L. Wang Y. Huang D. Zhang T. (2016). Antibiotic resistance genes and human bacterial pathogens: co-occurrence, removal, and enrichment in municipal sewage sludge digesters. *Water Res.* 91 1–10. 10.1016/j.watres.2015.11.071 26773390

[B25] Katoh K. Standley D. M. (2013). MAFFT multiple sequence alignment software version 7: improvements in performance and usability. *Mol. Biol. Evol.* 30 772–780. 10.1093/molbev/mst010 23329690 PMC3603318

[B26] Khan S. Beattie T. K. Knapp C. W. (2016). Relationship between antibiotic- and disinfectant-resistance profiles in bacteria harvested from tap water. *Chemosphere* 152 132–141. 10.1016/j.chemosphere.2016.02.086 26966812

[B27] Kumar M. Ram B. Honda R. Poopipattana C. Vu Duc C. Chaminda T. (2019). Concurrence of antibiotic resistant bacteria (ARB), viruses, pharmaceuticals and personal care products (PPCPs) in ambient waters of Guwahati, India: Urban vulnerability and resilience perspective. *Sci. Total Environ.* 693:133640. 10.1016/j.scitotenv.2019.133640 31377355

[B28] Labella A. Molero R. Leiva-Rebollo R. Perez-Recuerda R. Borrego J. J. (2021). Identification, resistance to antibiotics and biofilm formation of bacterial strains isolated from a reverse osmosis system of a drinking water treatment plant. *Sci. Total Environ.* 774:145718. 10.1016/j.scitotenv.2021.145718

[B29] Li B. Ju F. Cai L. Zhang T. (2015). Profile and fate of bacterial pathogens in sewage treatment plants revealed by high-throughput metagenomic approach. *Environ. Sci. Technol.* 49 10492–10502. 10.1021/acs.est.5b02345 26252189

[B30] Li W. Y. Tan Q. W. Zhou W. Chen J. P. Li Y. Wang F. (2020). Impact of substrate material and chlorine/chloramine on the composition and function of a young biofilm microbial community as revealed by high-throughput 16S rRNA sequencing. *Chemosphere* 242:10. 10.1016/j.chemosphere.2019.125310 31896192

[B31] Lin H. Ye C. Chen S. Zhang S. Yu X. (2017). Viable but non-culturable E. coli induced by low level chlorination have higher persistence to antibiotics than their culturable counterparts. *Environ. Pollut.* 230 242–249. 10.1016/j.envpol.2017.06.047 28662489

[B32] Liu S.-S. Qu H.-M. Yang D. Hu H. Liu W.-L. Qiu Z.-G. (2018). Chlorine disinfection increases both intracellular and extracellular antibiotic resistance genes in a full-scale wastewater treatment plant. *Water Res.* 136 131–136. 10.1016/j.watres.2018.02.036 29501757

[B33] Luo L.-W. Wu Y.-H. Yu T. Wang Y.-H. Chen G.-Q. Tong X. (2021). Evaluating method and potential risks of chlorine-resistant bacteria (CRB): a review. *Water Res.* 188:116474. 10.1016/j.watres.2020.116474 33039832

[B34] Ma L. Li B. Zhang T. (2019). New insights into antibiotic resistome in drinking water and management perspectives: a metagenomic based study of small-sized microbes. *Water Res.* 152 191–201. 10.1016/j.watres.2018.12.069 30669041

[B35] Ma L. Li B. Jiang X.-T. Wang Y.-L. Xia Y. Li A.-D. (2017). Catalogue of antibiotic resistome and host-tracking in drinking water deciphered by a large scale survey. *Microbiome* 5:154. 10.1186/s40168-017-0369-0 29179769 PMC5704573

[B36] Mao Y. Xia Y. Wang Z. Zhang T. (2014). Reconstructing a thauera genome from a hydrogenotrophic-denitrifying consortium using metagenomic sequence data. *Appl. Microbiol. Biotechnol.* 98 6885–6895. 10.1007/s00253-014-5756-x 24769905

[B37] Masoud S. M. Abd El-Baky R. M. Aly S. A. Ibrahem R. A. (2021). Co-Existence of certain ESBLs, MBLs and plasmid mediated quinolone resistance genes among MDR *E. coli* isolated from different clinical specimens in egypt. *Antibiot. Basel* 10:835. 10.3390/antibiotics10070835 34356756 PMC8300665

[B38] Massa S. Caruso M. Trovatelli F. Tosques M. (1998). Comparison of plate count agar and R2A medium for enumeration of heterotrophic bacteria in natural mineral water. *World J. Microbiol. Biotechnol.* 14 727–730. 10.1023/A:1008893627877

[B39] Mas-Ud M. A. Ali R. Hasan Z. (2020). Molecular detection and biological control of human hair dandruff causing microorganism *Staphylococcus aureus*. *J. Pure Appl. Microbiol.* 14 147–156. 10.22207/JPAM.14.1.16

[B40] Mazel D. (2006). Integrons: agents of bacterial evolution. *Nat. Rev. Microbiol.* 4 608–620. 10.1038/nrmicro1462 16845431

[B41] Mombini S. Rezatofighi S. E. Kiyani L. Motamedi H. (2019). Diversity and metallo-beta-lactamase-producing genes in *Pseudomonas aeruginosa* strains isolated from filters of household water treatment systems. *J. Environ. Manag.* 231 413–418. 10.1016/j.jenvman.2018.10.068 30368151

[B42] Mounaouer B. Abdennaceur H. (2016). Modeling and kinetic characterization of wastewater disinfection using chlorine and UV irradiation. *Environ. Sci. Pollut. Res.* 23 19861–19875. 10.1007/s11356-016-7173-4 27421857

[B43] Nielsen M. C. Jiang S. C. (2019). Alterations of the human skin microbiome after ocean water exposure. *Mar. Pollut. Bull.* 145 595–603. 10.1016/j.marpolbul.2019.06.047 31590829 PMC8061468

[B44] Oh S. Hammes F. Liu W. T. (2018). Metagenomic characterization of biofilter microbial communities in a full-scale drinking water treatment plant. *Water Res.* 128 278–285. 10.1016/j.watres.2017.10.054 29107912

[B45] Perez-Vidal A. Patricia Rivera-Sanchez S. Janeth Florez-Elvira L. Antonio Silva-Leal J. Diaz-Gomez J. Fernanda Herrera-Cuero L. (2019). Removal of E. coli and *Salmonella* in pot ceramic filters operating at different filtration rates. *Water Res.* 159 358–364. 10.1016/j.watres.2019.05.028 31112888

[B46] Poghosyan L. Koch H. Frank J. Van Kessel M. a. H. J. Cremers G. Van Alen T. (2020). Metagenomic profiling of ammonia- and methane-oxidizing microorganisms in two sequential rapid sand filters. *Water Res.* 185:116288. 10.1016/j.watres.2020.116288 32810745

[B47] Potgieter S. C. Dai Z. Venter S. N. Sigudu M. Pinto A. J. (2020). Microbial nitrogen metabolism in chloraminated drinking water reservoirs. *Msphere* 5:2. 10.1128/mSphere.00274-20 32350093 PMC7193043

[B48] Pruden A. Pei R. Storteboom H. Carlson K. H. (2006). Antibiotic resistance genes as emerging contaminants: studies in northern colorado. *Environ. Sci. Technol.* 40 7445–7450. 10.1021/es060413l 17181002

[B49] Pu Q. Zhao L.-X. Li Y.-T. Su J.-Q. (2020). Manure fertilization increase antibiotic resistance in soils from typical greenhouse vegetable production bases, China. *J. Hazard. Mater.* 391:122267. 10.1016/j.jhazmat.2020.122267 32062545

[B50] Qian X. Gu J. Sun W. Wang X.-J. Su J.-Q. Stedfeld R. (2018). Diversity, abundance, and persistence of antibiotic resistance genes in various types of animal manure following industrial composting. *J. Hazard. Mater.* 344 716–722. 10.1016/j.jhazmat.2017.11.020 29154097

[B51] Shekhawat S. S. Gupta A. B. Kulshreshtha N. M. Prakash R. (2021). UV disinfection studies on chlorine tolerant bacteria recovered from treated sewage. *J. Environ. Chem. Eng.* 9:105253. 10.1016/j.jece.2021.105253

[B52] Shekhawat S. S. Kulshreshtha N. M. Gupta A. B. (2020). Investigation of chlorine tolerance profile of dominant gram negative bacteria recovered from secondary treated wastewater in Jaipur, India. *J. Environ. Manag.* 255:109827. 10.1016/j.jenvman.2019.109827 31739205

[B53] Su H.-C. Liu Y.-S. Pan C.-G. Chen J. He L.-Y. Ying G.-G. (2018). Persistence of antibiotic resistance genes and bacterial community changes in drinking water treatment system: from drinking water source to tap water. *Sci. Total Environ.* 616 453–461. 10.1016/j.scitotenv.2017.10.318 29127799

[B54] Su J.-Q. Wei B. Ou-Yang W.-Y. Huang F.-Y. Zhao Y. Xu H.-J. (2015). Antibiotic resistome and its association with bacterial communities during sewage sludge composting. *Environ. Sci. Technol.* 49 7356–7363. 10.1021/acs.est.5b01012 26018772

[B55] Sun Y. M. Guo G. Tian F. Chen H. H. Liu W. J. Li M. (2021). Antibiotic resistance genes and bacterial community on the surfaces of five cultivars of fresh tomatoes. *Ecotoxicology* 30 1550–1558. 10.1007/s10646-020-02303-3 33184734

[B56] Torkar K. G. Drazetic M. (2017). The microbial contamination and the presence of beta-lactamase producing Gram-negative bacteria in the water and on the surfaces of public recreation water facilities. *Int. J. Environ. Health Res.* 27 293–305. 10.1080/09603123.2017.1342227 28631499

[B57] Ullmann I. F. Tunsjo H. S. Andreassen M. Nielsen K. M. Lund V. Charnock C. (2019). Detection of aminoglycoside resistant bacteria in sludge samples from norwegian drinking water treatment plants. *Front. Microbiol.* 10:487. 10.3389/fmicb.2019.00487 30918503 PMC6424899

[B58] Wan K. Lin W. Zhu S. Zhang S. Yu X. (2020). Biofiltration and disinfection codetermine the bacterial antibiotic resistome in drinking water: a review and meta-analysis. *Front. Environ. Sci. Eng.* 14:10. 10.1007/s11783-019-1189-1

[B59] Wang J. H. Lu J. Zhang Y. X. Wu J. Luo Y. M. Liu H. (2018). Metagenomic analysis of antibiotic resistance genes in coastal industrial mariculture systems. *Biores. Technol.* 253 235–243. 10.1016/j.biortech.2018.01.035 29353751

[B60] Wang S. Ma X. Liu Y. Yi X. Du G. Li J. (2020). Fate of antibiotics, antibiotic-resistant bacteria, and cell-free antibiotic-resistant genes in full-scale membrane bioreactor wastewater treatment plants. *Bioresour. Technol.* 302:122825. 10.1016/j.biortech.2020.122825 31986335

[B61] Wu D.-L. Zhang M. He L.-X. Zou H.-Y. Liu Y.-S. Li B.-B. (2020). Contamination profile of antibiotic resistance genes in ground water in comparison with surface water. *Sci. Total Environ.* 715:136975. 10.1016/j.scitotenv.2020.136975 32018106

[B62] Xu L. Campos L. C. Canales M. Ciric L. (2020). Drinking water biofiltration: Behaviour of antibiotic resistance genes and the association with bacterial community. *Water Res.* 182:115954. 10.1016/j.watres.2020.115954 32650149

[B63] Xu L. Ouyang W. Qian Y. Su C. Su J. Chen H. (2016). High-throughput profiling of antibiotic resistance genes in drinking water treatment plants and distribution systems. *Environ. Pollut.* 213 119–126. 10.1016/j.envpol.2016.02.013 26890482

[B64] Xu R. Yang Z. H. Wang Q. P. Bai Y. Liu J. B. Zheng Y. (2018). Rapid startup of thermophilic anaerobic digester to remove tetracycline and sulfonamides resistance genes from sewage sludge. *Sci. Total Environ.* 612 788–798. 10.1016/j.scitotenv.2017.08.295 28866406

[B65] Yin X. Jiang X.-T. Chai B. Li L. Yang Y. Cole J. R. (2018). ARGs-OAP v2.0 with an expanded SARG database and Hidden Markov Models for enhancement characterization and quantification of antibiotic resistance genes in environmental metagenomes. *Bioinformatics* 34 2263–2270. 10.1093/bioinformatics/bty053 29408954

[B66] Zeng Q. Sun J. Zhu L. (2019). Occurrence and distribution of antibiotics and resistance genes in greenhouse and open-field agricultural soils in China. *Chemosphere* 224 900–909. 10.1016/j.chemosphere.2019.02.167 30986896

[B67] Zhang G. Lu S. Wang Y. Liu X. Liu Y. Xu J. (2020b). Occurrence of antibiotics and antibiotic resistance genes and their correlations in lower Yangtze River, China. *Environ. Pollut.* 257:113365. 10.1016/j.envpol.2019.113365 31818612

[B68] Zhang H. Zhang Q. Song J. Zhang Z. Chen S. Long Z. (2020c). Tracking resistomes, virulence genes, and bacterial pathogens in long-term manure-amended greenhouse soils. *J. Hazard. Mater.* 396:122618. 10.1016/j.jhazmat.2020.122618 32298867

[B69] Zhang K. Xin R. Zhao Z. Ma Y. Zhang Y. Niu Z. (2020d). Antibiotic resistance genes in drinking water of China: occurrence, distribution and influencing factors. *Ecotoxicol. Environ. Safety* 188:109837. 10.1016/j.ecoenv.2019.109837 31683044

[B70] Zhang S. Abbas M. Rehman M. U. Huang Y. Zhou R. Gong S. (2020e). Dissemination of antibiotic resistance genes (ARGs) via integrons in *Escherichia coli*: a risk to human health. *Environ. Pollut.* 266:115260. 10.1016/j.envpol.2020.115260 32717638

[B71] Zhang G. Guan Y. Zhao R. Feng J. Huang J. Ma L. (2020a). Metagenomic and network analyses decipher profiles and co-occurrence patterns of antibiotic resistome and bacterial taxa in the reclaimed wastewater distribution system. *J. Hazard. Mater.* 400:123170. 10.1016/j.jhazmat.2020.123170 32590136

[B72] Zhang H. C. Chang F. Y. Shi P. Ye L. Zhou Q. Pan Y. (2019). Antibiotic Resistome alteration by different disinfection strategies in a full-scale drinking water treatment plant deciphered by metagenomic assembly. *Environ. Sci. Technol.* 53 2141–2150. 10.1021/acs.est.8b05907 30673217

[B73] Zhang L. Gu J. Wang X. Zhang R. Tuo X. Guo A. (2018). Fate of antibiotic resistance genes and mobile genetic elements during anaerobic co-digestion of Chinese medicinal herbal residues and swine manure. *Bioresour. Technol.* 250 799–805. 10.1016/j.biortech.2017.10.100 30001586

[B74] Zhang S. Yang G. Hou S. Zhang T. Li Z. Liang F. (2018). Distribution of ARGs and MGEs among glacial soil, permafrost, and sediment using metagenomic analysis. *Environ. Pollut.* 234 339–346. 10.1016/j.envpol.2017.11.031 29195175

[B75] Zhang S. Wang Y. Lu J. Yu Z. Song H. Bond P. L. (2021). Chlorine disinfection facilitates natural transformation through ROS-mediated oxidative stress. *Isme J.* 15 2969–2985. 10.1038/s41396-021-00980-4 33941886 PMC8091644

[B76] Zhao Q. Q. He H. Gao K. Li T. Dong B. Z. (2022). Fate, mobility, and pathogenicity of drinking water treatment plant resistomes deciphered by metagenomic assembly and network analyses. *Sci. Total Environ.* 804:150095. 10.1016/j.scitotenv.2021.150095 34509829

[B77] Zhou Z. Xu L. Zhu L. Liu Y. Shuai X. Lin Z. (2021). Metagenomic analysis of microbiota and antibiotic resistome in household activated carbon drinking water purifiers. *Environ. Int.* 148 106394–106394. 10.1016/j.envint.2021.106394 33486296

[B78] Zhu L. Zhao Y. Yang K. Chen J. Zhou H. Chen X. (2019). Host bacterial community of MGEs determines the risk of horizontal gene transfer during composting of different animal manures. *Environ. Pollut.* 250 166–174. 10.1016/j.envpol.2019.04.037 30995570

[B79] Zhu N. J. Ghosh S. Edwards M. A. Pruden A. (2021). Interplay of biologically active carbon filtration and chlorine-based disinfection in mitigating the dissemination of antibiotic resistance genes in water reuse distribution systems. *Environ. Sci. Technol.* 55 8329–8340. 10.1021/acs.est.1c01199 34080846

