# Supplementary material for: Characteristics of Antibiotic Resistance Genes and Antibiotic-Resistant Bacteria in Full-Scale Drinking Water Treatment System Using Metagenomics and Culturing
Source: Front Microbiol. 2022 Feb 22;12:798442. doi: 10.3389/fmicb.2021.798442 (PMC8902363; doi:10.3389/fmicb.2021.798442)
Supplement: Supplementary file 8 [file Data_Sheet_8.docx]

***Supplementary Material***

**Characteristics of antibiotic resistance genes (ARGs) and antibiotic resistant bacteria (ARB) in full-scale drinking water treatment system using metagenomics and culturing**

Qihui Gu^1#^,Ming Sun^1#^,Tao Lin^1^, Youxiong Zhang^1^,Xianhu Wei^1^, Shi Wu^1^, Shuhong Zhang^1^, Rui Pang^1^, Juan Wang^2^, Yu Ding^1^, Zhenjie Liu^1^, Ling Chen^1^, Wei Chen^1^, Xiuhua Lin^1^, Jumei Zhang^1^, Moutong Chen^1^, Liang Xue^1^,Qingping Wu^1^*

1.Guangdong Provincial Key Laboratory of Microbial Safety and Health, State Key Laboratory of Applied Microbiology Southern China, Institute  of  Microbiology, Guangdong Academy of Sciences, 510070, PR China

2. College of Food Science, South China Agricultural University, Guangzhou 510640, P.R. China

# These authors contributed equally.

* Corresponding author: Qingping Wu

Tel./fax: 86-020-83911498

E-mail address: wuqp203@163.com

1. Supplementary Figures


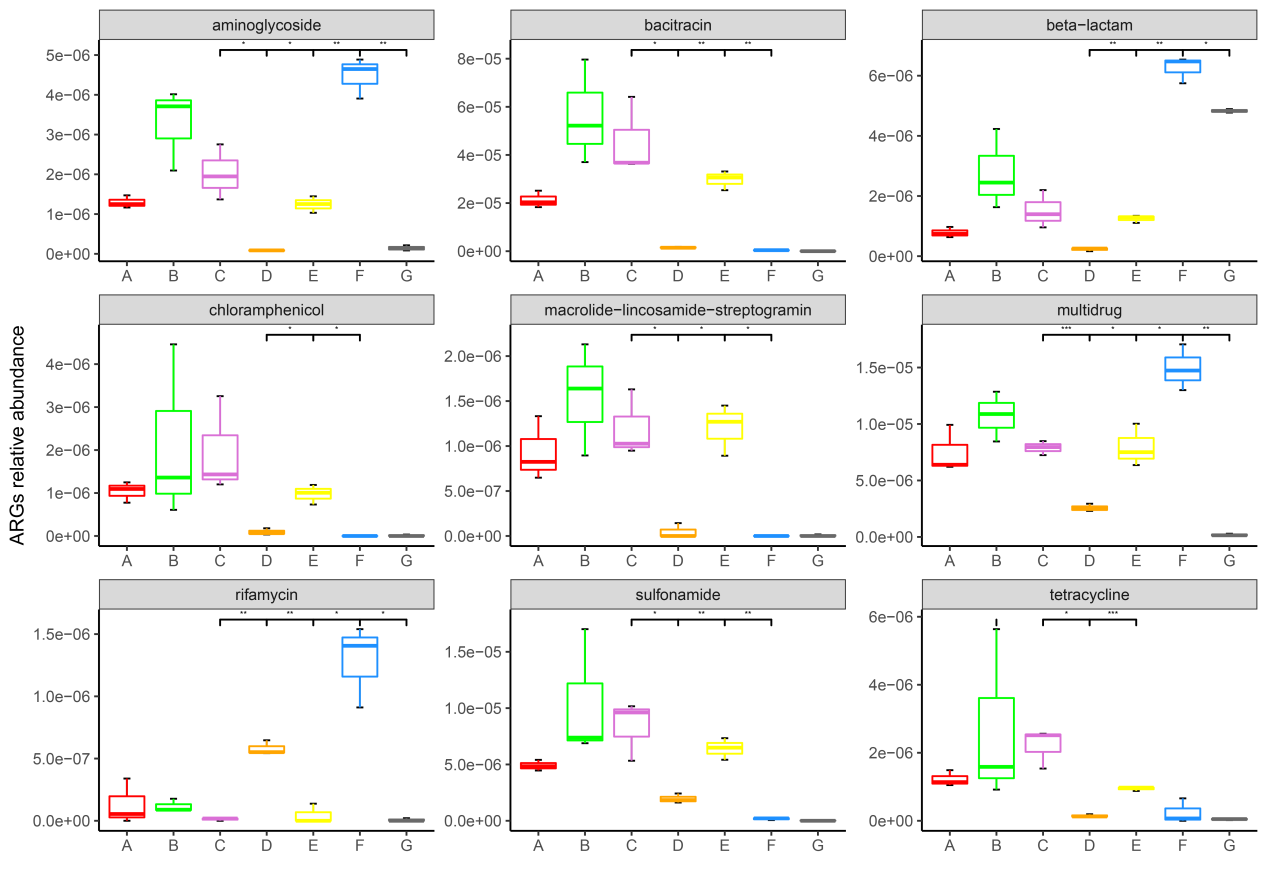


Fig.S1 ARGs relative abundance across drinking water treatment processes.


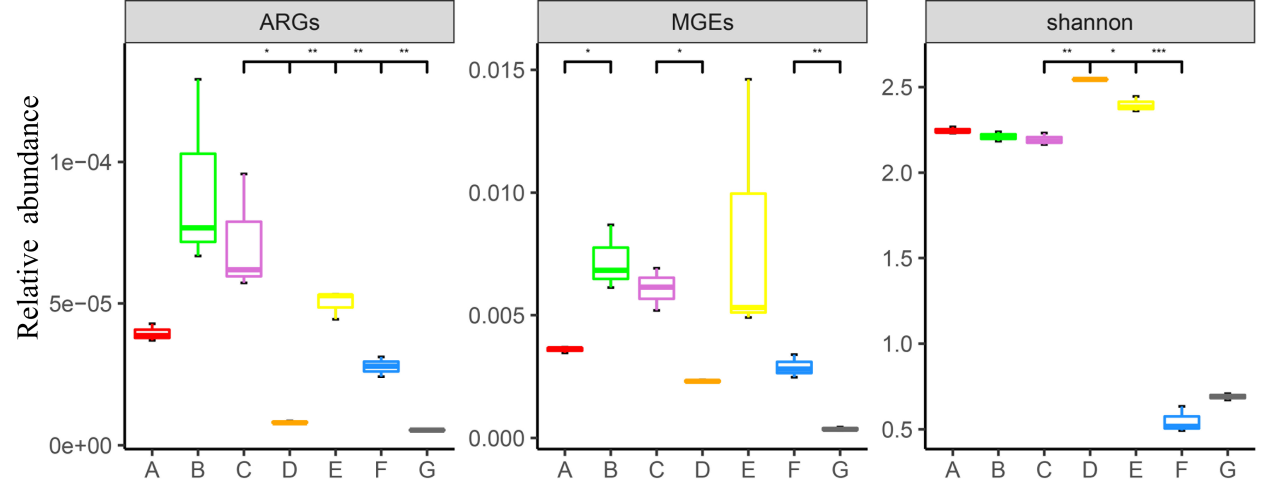


Fig.S2 KW test of ARGs, MGEs, and Shannon index in drinking water treatment processes


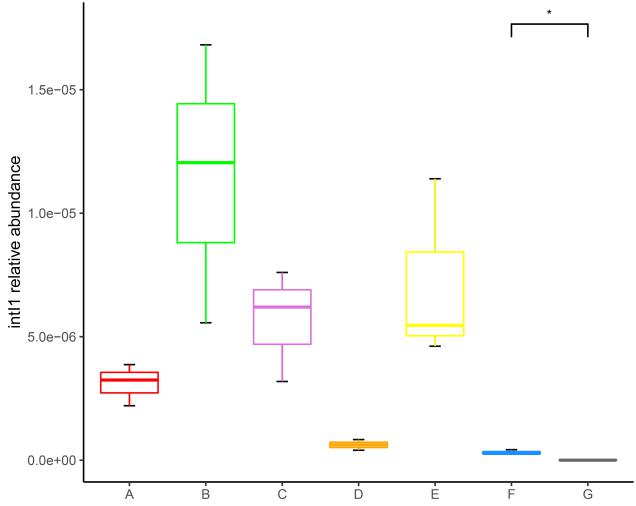


Fig.S3 *Intl*1 relative abundance across drinking water treatment processes.


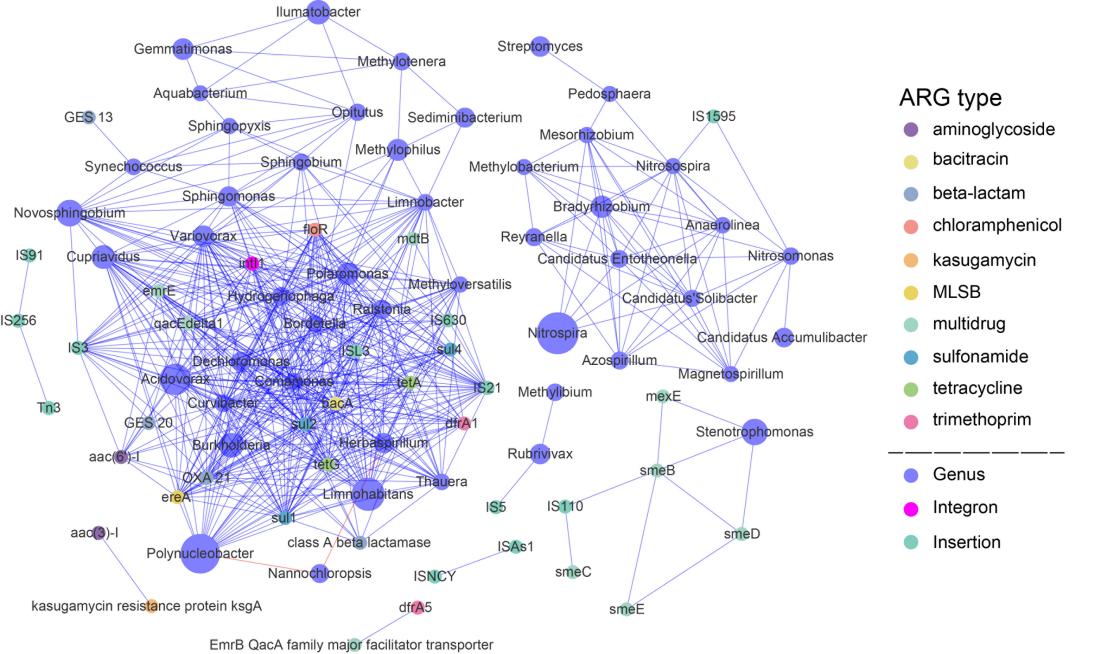


Fig.S4 Network analysis revealed the co-occurrence patterns of ARG subtypes, MGEs, and bacterial taxa with significant Spearman’s correlation coefficient≥0.8 (p < 0.01) across all water samples in the DWST. The nodes were colored according to network modularity and node size is proportional to the number of connections to other nodes (degree).


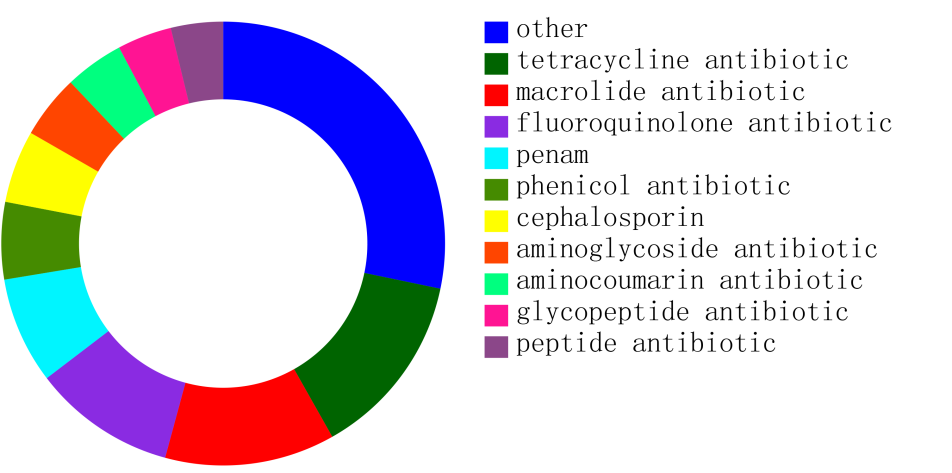


Fig.S5 ARG types circle map of strain F2

1. Supplementary Tables

Table S1 Basic information of the metagenomic datasets in this study

| Sample | | Raw dataset  read number | Clean dataset  read number | Clean Bases |
| --- | --- | --- | --- | --- |
| A | A_1 | 71827458 | 62310664 | 9255377926 |
|  | A_2 | 78825524 | 67535218 | 10036115480 |
|  | A_3 | 88177092 | 74432942 | 11068035951 |
| B | B_1 | 82918126 | 70959362 | 10525477397 |
|  | B_2 | 70735810 | 60943442 | 9035196113 |
|  | B_3 | 83597552 | 71471214 | 10595341444 |
| C | C_1 | 76866016 | 65870054 | 9772964835 |
|  | C_2 | 106347036 | 46069690 | 6842359564 |
|  | C_3 | 82561420 | 70596950 | 10487510665 |
| D | D_1 | 80027272 | 68324980 | 10133781087 |
|  | D_2 | 69411234 | 59535770 | 8821061553 |
|  | D_3 | 86175766 | 73788162 | 10948791550 |
| E | E_1 | 70715224 | 60860456 | 9044593583 |
|  | E_2 | 73575094 | 63368322 | 9404788594 |
|  | E_3 | 83606470 | 72217360 | 10723526205 |
| F | F_1 | 78234790 | 63982358 | 9124939182 |
|  | F_2 | 73003308 | 60898148 | 8737112715 |
|  | F_3 | 69418168 | 58468290 | 8381477682 |
| G | G_1 | 88756178 | 75263056 | 11117325686 |
|  | G_2 | 82613422 | 69905704 | 10308937597 |
|  | G_3 | 80572816 | 68701650 | 10129564803 |

Table S2 Comparison of phylogenetic taxonomic affiliation in different sampling sites

| Cluster | A1 | A2 | A3 | B1 | B2 | B3 | C1 | C2 | C3 | D1 | D2 | D3 | E1 | E2 | E3 | F1 | F2 | F3 | G1 | G2 | G3 |
| --- | --- | --- | --- | --- | --- | --- | --- | --- | --- | --- | --- | --- | --- | --- | --- | --- | --- | --- | --- | --- | --- |
| *Acidovorax* | 21 | 18 | 17 | 27 | 26 | 21 | 21 | 20 | 21 | 2 | 2 | 2 | 17 | 18 | 19 | 0 | 0 | 0 | 0 | 0 | 0 |
| *Acinetobacter* | 2 | 2 | 2 | 2 | 2 | 2 | 2 | 1 | 1 | 1 | 1 | 2 | 2 | 2 | 2 | 1 | 2 | 1 | 1 | 1 | 0 |
| *Enterobacter* | 1 | 1 | 0 | 1 | 0 | 1 | 0 | 1 | 1 | 0 | 0 | 0 | 1 | 1 | 1 | 0 | 0 | 0 | 0 | 0 | 0 |
| *Escherichia* | 3 | 3 | 5 | 5 | 4 | 3 | 2 | 4 | 4 | 2 | 2 | 2 | 4 | 3 | 3 | 3 | 4 | 2 | 1 | 3 | 1 |
| *Herbaspirillum* | 0 | 1 | 1 | 1 | 1 | 0 | 1 | 1 | 0 | 0 | 0 | 0 | 1 | 1 | 1 | 0 | 0 | 0 | 0 | 0 | 0 |
| *Klebsiella* | 0 | 4 | 2 | 2 | 5 | 4 | 3 | 2 | 5 | 1 | 0 | 0 | 4 | 3 | 4 | 1 | 1 | 1 | 1 | 0 | 0 |
| *Limnohabitans* | 0 | 2 | 3 | 2 | 3 | 0 | 2 | 2 | 2 | 0 | 0 | 0 | 3 | 2 | 2 | 0 | 0 | 0 | 0 | 0 | 0 |
| *Mycobacterium* | 0 | 0 | 1 | 0 | 0 | 0 | 0 | 0 | 0 | 0 | 0 | 0 | 0 | 0 | 0 | 3 | 3 | 2 | 0 | 0 | 0 |
| *Nitrosomonas* | 0 | 0 | 0 | 0 | 1 | 0 | 1 | 1 | 1 | 0 | 1 | 1 | 0 | 1 | 1 | 0 | 0 | 0 | 0 | 0 | 0 |
| *Nitrosospira* | 0 | 0 | 0 | 0 | 0 | 0 | 0 | 0 | 0 | 1 | 1 | 1 | 0 | 0 | 0 | 0 | 0 | 0 | 0 | 0 | 0 |
| *Paenibacillus* | 0 | 0 | 0 | 0 | 0 | 0 | 0 | 0 | 0 | 0 | 0 | 0 | 0 | 0 | 0 | 0 | 0 | 0 | 1 | 0 | 0 |
| *Polaromonas* | 4 | 4 | 4 | 5 | 5 | 3 | 4 | 5 | 5 | 1 | 0 | 0 | 4 | 5 | 5 | 0 | 0 | 0 | 0 | 0 | 0 |
| *Polynucleobacter* | 11 | 13 | 11 | 14 | 16 | 8 | 14 | 17 | 18 | 1 | 0 | 0 | 12 | 10 | 16 | 1 | 2 | 0 | 0 | 0 | 0 |
| *Pseudomonas* | 10 | 7 | 7 | 9 | 11 | 9 | 11 | 10 | 11 | 4 | 3 | 4 | 9 | 9 | 10 | 3 | 3 | 3 | 0 | 1 | 0 |
| *Ramlibacter* | 1 | 0 | 1 | 1 | 1 | 0 | 1 | 1 | 1 | 0 | 1 | 0 | 1 | 1 | 1 | 0 | 0 | 0 | 0 | 0 | 0 |
| *Rhodococcus* | 0 | 1 | 1 | 1 | 0 | 0 | 1 | 0 | 1 | 0 | 0 | 0 | 0 | 0 | 0 | 2 | 2 | 2 | 0 | 0 | 1 |
| *Rubrivivax* | 0 | 1 | 1 | 1 | 1 | 1 | 0 | 0 | 0 | 1 | 1 | 1 | 1 | 1 | 0 | 0 | 0 | 0 | 0 | 0 | 0 |
| *Salmonella* | 0 | 0 | 0 | 0 | 0 | 0 | 0 | 1 | 0 | 0 | 0 | 0 | 0 | 0 | 0 | 0 | 0 | 0 | 0 | 0 | 0 |
| *Stenotrophomonas* | 9 | 8 | 15 | 14 | 13 | 14 | 13 | 12 | 14 | 0 | 0 | 1 | 14 | 13 | 12 | 15 | 15 | 15 | 10 | 6 | 7 |
| Unclassified | 153 | 152 | 151 | 199 | 219 | 139 | 192 | 184 | 186 | 50 | 34 | 67 | 150 | 158 | 178 | 13 | 18 | 12 | 12 | 1 | 6 |
| *Variovorax* | 1 | 1 | 1 | 1 | 1 | 1 | 1 | 1 | 1 | 0 | 0 | 0 | 1 | 1 | 1 | 0 | 1 | 0 | 0 | 0 | 0 |

Table S3 Significant (P-value ≤ 0.01) and strong (Spearman’s r ≥ 0.8) correlations among ARG subtypes

| Source | Target | Correlation | pvlaue | p.adjust |
| --- | --- | --- | --- | --- |
| aminoglycoside__aac(3)-I | kasugamycin__kasugamycin-resistance-protein-ksgA | 1 | 0 | 0 |
| bacitracin__bacA | sulfonamide__sul1 | 0.963903 | 2.25E-12 | 5.43E-09 |
| chloramphenicol__floR | tetracycline__tetG | 0.947107 | 7.94E-11 | 1.91E-07 |
| multidrug__EmrB-QacA-family-  major-facilitator-transporter | trimethoprim__dfrA5 | 0.939171 | 2.90E-10 | 7.00E-07 |
| multidrug__qacEdelta1 | sulfonamide__sul1 | 0.934896 | 5.44E-10 | 1.31E-06 |
| sulfonamide__sul1 | sulfonamide__sul2 | 0.934568 | 5.70E-10 | 1.37E-06 |
| beta-lactam__OXA-21 | trimethoprim__dfrA1 | 0.931923 | 8.22E-10 | 1.98E-06 |
| bacitracin__bacA | beta-lactam__OXA-21 | 0.930574 | 9.85E-10 | 2.37E-06 |
| multidrug__smeB | multidrug__smeD | 0.929332 | 1.16E-09 | 2.79E-06 |
| chloramphenicol__floR | trimethoprim__dfrA1 | 0.923467 | 2.42E-09 | 5.82E-06 |
| beta-lactam__OXA-21 | chloramphenicol__floR | 0.922788 | 2.62E-09 | 6.31E-06 |
| beta-lactam__OXA-21 | macrolide-lincosamide-streptogramin__ereA | 0.922734 | 2.64E-09 | 6.34E-06 |
| sulfonamide__sul4 | tetracycline__tetA | 0.921142 | 3.18E-09 | 7.65E-06 |
| sulfonamide__sul1 | tetracycline__tetG | 0.919446 | 3.87E-09 | 9.29E-06 |
| chloramphenicol__floR | tetracycline__tetA | 0.919306 | 3.93E-09 | 9.44E-06 |
| chloramphenicol__floR | sulfonamide__sul2 | 0.918358 | 4.38E-09 | 1.05E-05 |
| bacitracin__bacA | sulfonamide__sul2 | 0.914696 | 6.54E-09 | 1.57E-05 |
| bacitracin__bacA | macrolide-lincosamide-streptogramin__ereA | 0.913874 | 7.14E-09 | 1.71E-05 |
| beta-lactam__OXA-21 | sulfonamide__sul1 | 0.913428 | 7.49E-09 | 1.80E-05 |
| chloramphenicol__floR | sulfonamide__sul4 | 0.911364 | 9.29E-09 | 2.23E-05 |
| beta-lactam__OXA-21 | tetracycline__tetG | 0.908997 | 1.18E-08 | 2.83E-05 |
| bacitracin__bacA | chloramphenicol__floR | 0.908729 | 1.21E-08 | 2.91E-05 |
| aminoglycoside__aac(6_)-I | bacitracin__bacA | 0.906758 | 1.48E-08 | 3.53E-05 |
| bacitracin__bacA | multidrug__qacEdelta1 | 0.906667 | 1.49E-08 | 3.56E-05 |
| beta-lactam__OXA-21 | sulfonamide__sul2 | 0.906124 | 1.57E-08 | 3.75E-05 |
| chloramphenicol__floR | sulfonamide__sul1 | 0.90567 | 1.64E-08 | 3.92E-05 |
| aminoglycoside__aac(6_)-I | macrolide-lincosamide-streptogramin__ereA | 0.902859 | 2.14E-08 | 5.12E-05 |
| macrolide-lincosamide-streptogramin__ereA | trimethoprim__dfrA1 | 0.901322 | 2.47E-08 | 5.91E-05 |
| macrolide-lincosamide-streptogramin__ereA | tetracycline__tetA | 0.900478 | 2.67E-08 | 6.38E-05 |
| sulfonamide__sul2 | sulfonamide__sul4 | 0.900198 | 2.74E-08 | 6.54E-05 |
| multidrug__smeB | multidrug__smeE | 0.900163 | 2.75E-08 | 6.56E-05 |
| macrolide-lincosamide-streptogramin__ereA | tetracycline__tetG | 0.896978 | 3.66E-08 | 8.73E-05 |
| multidrug__qacEdelta1 | sulfonamide__sul2 | 0.896609 | 3.78E-08 | 9.01E-05 |
| bacitracin__bacA | tetracycline__tetG | 0.896505 | 3.82E-08 | 9.09E-05 |
| multidrug__mexE | multidrug__smeB | 0.895631 | 4.12E-08 | 9.81E-05 |
| sulfonamide__sul2 | tetracycline__tetA | 0.894431 | 4.57E-08 | 0.000109 |
| bacitracin__bacA | multidrug__emrE | 0.891041 | 6.09E-08 | 0.000145 |
| macrolide-lincosamide-streptogramin__ereA | sulfonamide__sul2 | 0.889991 | 6.64E-08 | 0.000158 |
| sulfonamide__sul2 | tetracycline__tetG | 0.888646 | 7.41E-08 | 0.000176 |
| bacitracin__bacA | tetracycline__tetA | 0.888241 | 7.66E-08 | 0.000182 |
| sulfonamide__sul4 | trimethoprim__dfrA1 | 0.887268 | 8.28E-08 | 0.000197 |
| multidrug__smeD | multidrug__smeE | 0.886581 | 8.75E-08 | 0.000208 |
| tetracycline__tetA | trimethoprim__dfrA1 | 0.884683 | 1.02E-07 | 0.000241 |
| multidrug__emrE | sulfonamide__sul1 | 0.883483 | 1.12E-07 | 0.000265 |
| chloramphenicol__floR | macrolide-lincosamide-streptogramin__ereA | 0.880904 | 1.36E-07 | 0.000323 |
| tetracycline__tetG | trimethoprim__dfrA1 | 0.880458 | 1.41E-07 | 0.000333 |
| tetracycline__tetA | tetracycline__tetG | 0.880378 | 1.42E-07 | 0.000335 |
| beta-lactam__GES-20 | sulfonamide__sul4 | 0.876468 | 1.89E-07 | 0.000448 |
| aminoglycoside__aac(6_)-I | multidrug__qacEdelta1 | 0.875415 | 2.04E-07 | 0.000483 |
| bacitracin__bacA | sulfonamide__sul4 | 0.874773 | 2.14E-07 | 0.000506 |
| bacitracin__bacA | trimethoprim__dfrA1 | 0.873975 | 2.26E-07 | 0.000535 |
| multidrug__emrE | sulfonamide__sul2 | 0.872589 | 2.50E-07 | 0.00059 |
| macrolide-lincosamide-streptogramin__ereA | sulfonamide__sul4 | 0.872148 | 2.58E-07 | 0.000609 |
| aminoglycoside__aac(6_)-I | beta-lactam__GES-20 | 0.870332 | 2.92E-07 | 0.000691 |
| macrolide-lincosamide-streptogramin__ereA | sulfonamide__sul1 | 0.869963 | 3.00E-07 | 0.000708 |
| aminoglycoside__aac(6_)-I | sulfonamide__sul4 | 0.868539 | 3.31E-07 | 0.00078 |
| beta-lactam__class-A-beta-lactamase | sulfonamide__sul1 | 0.867523 | 3.54E-07 | 0.000836 |
| sulfonamide__sul1 | tetracycline__tetA | 0.865625 | 4.02E-07 | 0.000949 |
| aminoglycoside__aac(6_)-I | sulfonamide__sul1 | 0.86555 | 4.04E-07 | 0.000953 |
| aminoglycoside__aac(6_)-I | multidrug__emrE | 0.862737 | 4.87E-07 | 0.001147 |
| bacitracin__bacA | beta-lactam__class-A-beta-lactamase | 0.862735 | 4.87E-07 | 0.001147 |
| multidrug__emrE | tetracycline__tetG | 0.862707 | 4.88E-07 | 0.001148 |
| aminoglycoside__aac(6_)-I | beta-lactam__OXA-21 | 0.862327 | 5.00E-07 | 0.001176 |
| sulfonamide__sul1 | sulfonamide__sul4 | 0.85973 | 5.91E-07 | 0.001389 |
| sulfonamide__sul2 | trimethoprim__dfrA1 | 0.857639 | 6.74E-07 | 0.001585 |
| beta-lactam__OXA-21 | tetracycline__tetA | 0.857598 | 6.76E-07 | 0.001588 |
| beta-lactam__class-A-beta-lactamase | sulfonamide__sul2 | 0.856209 | 7.37E-07 | 0.001731 |
| polymyxin__arnA | quinolone__qepA | 0.852397 | 9.30E-07 | 0.002184 |
| aminoglycoside__aac(6_)-I | trimethoprim__dfrA1 | 0.850958 | 1.01E-06 | 0.00238 |
| multidrug__emrE | multidrug__qacEdelta1 | 0.849773 | 1.09E-06 | 0.002552 |
| beta-lactam__OXA-21 | multidrug__qacEdelta1 | 0.848565 | 1.17E-06 | 0.002739 |
| macrolide-lincosamide-streptogramin__mphA | multidrug__mdtB | 0.847728 | 1.23E-06 | 0.002876 |
| beta-lactam__GES-20 | multidrug__emrE | 0.844703 | 1.46E-06 | 0.003422 |
| aminoglycoside__aac(6_)-II | macrolide-lincosamide-streptogramin__mphA | 0.844056 | 1.52E-06 | 0.003549 |
| beta-lactam__OXA-21 | multidrug__mdtB | 0.843609 | 1.55E-06 | 0.003639 |
| multidrug__mexE | multidrug__smeE | 0.843275 | 1.58E-06 | 0.003706 |
| sulfonamide__sul1 | trimethoprim__dfrA1 | 0.839618 | 1.94E-06 | 0.004543 |
| beta-lactam__class-A-beta-lactamase | chloramphenicol__floR | 0.839203 | 1.99E-06 | 0.004647 |
| beta-lactam__OXA-21 | sulfonamide__sul4 | 0.838133 | 2.11E-06 | 0.004925 |
| chloramphenicol__floR | trimethoprim__dfrA5 | 0.83812 | 2.11E-06 | 0.004926 |
| aminoglycoside__aac(6_)-I | tetracycline__tetG | 0.838007 | 2.12E-06 | 0.004954 |
| beta-lactam__OXA-21 | multidrug__emrE | 0.835297 | 2.46E-06 | 0.005733 |
| sulfonamide__sul4 | tetracycline__tetG | 0.834051 | 2.63E-06 | 0.006125 |
| beta-lactam__GES-20 | chloramphenicol__catB | 0.83374 | 2.67E-06 | 0.006224 |
| multidrug__qacEdelta1 | sulfonamide__sul4 | 0.833236 | 2.74E-06 | 0.006389 |
| macrolide-lincosamide-streptogramin__ereA | multidrug__qacEdelta1 | 0.83118 | 3.05E-06 | 0.007115 |
| bacitracin__bacA | beta-lactam__GES-20 | 0.831105 | 3.07E-06 | 0.007139 |
| aminoglycoside__aac(6_)-I | tetracycline__tetA | 0.829353 | 3.36E-06 | 0.007814 |
| multidrug__mdtB | sulfonamide__sul2 | 0.825 | 4.19E-06 | 0.009745 |
| beta-lactam__GES-20 | sulfonamide__sul2 | 0.824524 | 4.29E-06 | 0.009976 |

Table S4 General characteristics of MAGs recovered from DWTS metagenomes

| MAG number | Completeness(%) | Contamination(%) | Classification |
| --- | --- | --- | --- |
| bin.405 | 60.15 | 1.7 | f__Gemmataceae; |
| bin.219 | 90.52 | 3.48 | s__UBA969 |
| bin.63 | 98.2 | 9.8 | f__Sphingobacteriaceae |
| Bin.520 | 53 | 9.84 | k__Bacteria |
| Bin.138 | 99.5 | 1.65 | s__Rhodococcus hoagii |
| Bin.84 | 93.64 | 3.26 | s__Rhodococcus erythropolis_D |
| Bin.162 | 55.36 | 7.32 | k__Bacteria |
| Bin.510 | 56.93 | 4.88 | g__Nitrospira_D |
| Bin.368 | 52.27 | 6.59 | k__Bacteria |
| Bin.542 | 55.42 | 2.19 | g__Fonsibacter |
| Bin.617 | 55.35 | 8.72 | k__Bacteria |
| Bin.291 | 57.56 | 7.02 | c__Alphaproteobacteria |
| Bin.358 | 62.16 | 5.14 | f__Beijerinckiaceae |
| Bin.71 | 68.97 | 2.74 | o__Rhizobiales |
| Bin.495 | 63.3 | 7.46 | f__Sphingomonadaceae |
| Bin.335 | 60.38 | 8 | o__Sphingomonadales |
| Bin.366 | 61.07 | 5.62 | o__Sphingomonadales |
| Bin.403 | 53.41 | 8.63 | p__Proteobacteria |
| Bin.320 | 90.15 | 9.94 | f__Steroidobacteraceae |
| Bin.195 | 95.51 | 2.02 | s__Stenotrophomonas maltophilia |
| Bin.210 | 90.58 | 9.56 | g__Pararheinheimera |
| Bin.407 | 96.17 | 4.78 | g__Perlucidibaca |
| Bin.394 | 92.26 | 3.26 | g__Perlucidibaca |
| Bin.306 | 66.71 | 6.33 | g__Nitrosomonas |
| Bin.469 | 81.35 | 9.1 | g__Methylotenera |
| Bin.326 | 83.09 | 7.73 | g__Methylophilus |
| Bin.278 | 96.55 | 9.09 | f__Burkholderiaceae |
| Bin.478 | 68.3 | 8.49 | f__Burkholderiaceae |
| Bin.262 | 89.19 | 9.7 | g__Limnobacter |
| Bin.373 | 67.72 | 6.87 | f__Burkholderiaceae |
| Bin.379 | 64.05 | 8.14 | f__Burkholderiaceae |
| Bin.442 | 56.05 | 8.61 | o__Burkholderiales |
| Bin.444 | 64.88 | 9.55 | o__Burkholderiales |

Table S5 ARG types and ARG subtypes distribution in MAGs recovered from DWTS metagenomes

| GeneID | Type | Subtype |
| --- | --- | --- |
| orf_2965\|Bin.84 | rifamycin | rifamycin__rifampinmonooxygenase |
| orf_7328\|Bin.84 | aminoglycoside | aminoglycoside__ant(2'')-I |
| orf_7330\|Bin.84 | aminoglycoside | aminoglycoside__ant(3'')-Ih-aac(6')-IId |
| orf_366\|Bin.195 | multidrug | multidrug__smeD |
| orf_367\|Bin.195 | multidrug | multidrug__smeE |
| orf_368\|Bin.195 | multidrug | multidrug__multidrug_transporter |
| orf_1151\|Bin.195 | beta-lactam | beta-lactam__metallo-beta-lactamase |
| orf_2411\|Bin.195 | aminoglycoside | aminoglycoside__aph(3')-IIb |
| orf_2522\|Bin.195 | multidrug | multidrug__mexE |
| orf_2523\|Bin.195 | multidrug | multidrug__smeB |
| orf_2524\|Bin.195 | multidrug | multidrug__smeC |
| orf_3597\|Bin.335 | chloramphenicol | chloramphenicol__floR |
| orf_2700\|Bin.379 | bacitracin | bacitracin__bacA |

Table S6. Species identified during drinking water treatment processes and sand filter based on culturing method.

| Isolates | Closest relative^a^ (accession no.) | Similarity (%) | Taxonomy |
| --- | --- | --- | --- |
|  | | | |
| A1 | *Tessaracoccus* sp. (FJ527820.1) | 99.85 | *Actinobacteria;Propionibacteriales;Propionibacteriaceae;Tessaracoccus; unclassified Tessaracoccus.* |
| A2 | *Bacillus* sp.(MT568620.1) | 100 | *Firmicutes; Bacilli; Bacillales; Bacillaceae; Bacillus.* |
| A3 | *Bacillus aryabhattai*(MT184818.1) | 100 | *Firmicutes; Bacilli; Bacillales; Bacillaceae; Priestia.* |
| A4 | *Paenibacillus* sp. (MK934382.1) | 99.86 | *Firmicutes; Bacilli; Bacillales; Paenibacillaceae; Paenibacillus.* |
| A5 | *Dickeya zeae* strain(KY817904.1) | 99.72 | *Proteobacteria; Gammaproteobacteria; Enterobacterales; Pectobacteriaceae; Dickeya.* |
| A6 | *Fictibacillus* sp.(MK757936.1) | 99.93 | *Firmicutes; Bacilli; Bacillales; Bacillaceae; Fictibacillus.* |
| B1 | *Chromobacterium rhizoryzae* (CP031968.1) | 100 | *Proteobacteria; Betaproteobacteria; Neisseriales; Chromobacteriaceae; Chromobacterium.* |
| B2 | *Hydrogenophaga laconesensis* (NR_149183.1) | 99.71 | *Proteobacteria; Betaproteobacteria; Burkholderiales; Comamonadaceae; Hydrogenophaga.* |
| B3 | *Pseudomonas aeruginosa* (MT646431.1) | 100 | *Proteobacteria; Gammaproteobacteria; Pseudomonadales; Pseudomonadaceae; Pseudomonas.* |
| B4 | *Hydrogenophaga laconesensis*(NR_149183.1) | 99.37 | *Proteobacteria; Betaproteobacteria; Burkholderiales; Comamonadaceae; Hydrogenophaga.* |
| B5 | *Hydrogenophaga laconesensis* (NR_149183.1) | 99.71 | *Proteobacteria; Betaproteobacteria; Burkholderiales; Comamonadaceae; Hydrogenophaga.* |
| B6 | *Moraxella* sp. (MT380814.1) | 100 | *Proteobacteria;Gammaproteobacteria;Pseudomonadales;Moraxellaceae;Moraxella;unclassified Moraxella.* |
| B7 | *Fictibacillus* sp. (MT541004.1) | 100 | *Firmicutes; Bacilli; Bacillales; Bacillaceae; Fictibacillus; unclassified Fictibacillus.* |
| B8 | *Bacillus altitudinis*(MT627439.1) | 100 | *Firmicutes; Bacilli; Bacillales; Bacillaceae; Bacillus.* |
| B9 | *Bacillus subtilis* (MN865922.1) | 100 | *Firmicutes; Bacilli; Bacillales; Bacillaceae; Bacillus.* |
| B10 | *Acidovorax* sp.(MN519578.1) | 100 | *Proteobacteria; Betaproteobacteria; Burkholderiales; Comamonadaceae; Acidovorax.* |
| B11 | *Pseudomonas aeruginosa*(MT109313.1) | 100 | *Proteobacteria; Gammaproteobacteria; Pseudomonadales; Pseudomonadaceae; Pseudomonas.* |
| B12 | *Hydrogenophaga laconesensis*(NR_149183.1) | 99.78 | *Proteobacteria; Betaproteobacteria; Burkholderiales; Comamonadaceae; Hydrogenophaga.* |
| B13 | *Pseudomonas aeruginosa* (NR_117678.1) | 100 | *Proteobacteria; Gammaproteobacteria; Pseudomonadales; Pseudomonadaceae; Pseudomonas.* |
| B14 | *Hydrogenophaga laconesensis*(NR_149183.1) | 99.70 | *Proteobacteria; Betaproteobacteria; Burkholderiales;Comamonadaceae; Hydrogenophaga.* |
| B15 | *Chromobacterium rhizoryzae*(NR_152068.1) | 99.70 | *Proteobacteria; Betaproteobacteria; Neisseriales; Chromobacteriaceae; Chromobacterium.* |
| B16 | *Aeromonas hydrophila*(NR_119190.1) | 99.92 | *Proteobacteria; Gammaproteobacteria; Aeromonadales; Aeromonadaceae; Aeromonas.* |
| B17 | *Enterobacter tabaci*(NR_146667.2) | 99.43 | *Proteobacteria; Gammaproteobacteria; Enterobacterales; Enterobacteriaceae; Enterobacter.* |
| B18 | *Bacillus aerius*(NR_118439.1) | 100 | *Firmicutes; Bacilli; Bacillales; Bacillaceae; Bacillus* |
| B19 | *Klebsiella pneumoniae* (FJ2 SC) | 100 | *Proteobacteria; Gammaproteobacteria;Enterobacterales;Enterobacteriaceae;Klebsiella.* |
| C1 | *Chromobacterium aquaticum*(LN995680.1) | 99.93 | *Proteobacteria; Betaproteobacteria; Neisseriales; Chromobacteriaceae; Chromobacterium.* |
| C2 | *Staphylococcus warneri* (MT072204.1) | 100 | *Firmicutes; Bacilli; Bacillales; Staphylococcaceae; Staphylococcus.* |
| C3 | *Bacillus toyonensis* ( MN543844.1) | 100 | *Firmicutes; Bacilli; Bacillales; Bacillaceae; Bacillus; Bacillus cereus group.* |
| C4 | *Bacillus cereus* (MN543837.1) | 100 | *Firmicutes; Bacilli; Bacillales; Bacillaceae; Bacillus; Bacillus cereus group.* |
| C5 | *Paenibacillus peoriae*(NR_042092.1) | 99.93 | *Firmicutes; Bacilli; Bacillales; Paenibacillaceae; Paenibacillus.* |
| C6 | *Bacillus aerophilu*s(MT102966.1) | 100 | *Firmicutes; Bacilli; Bacillales; Bacillaceae; Bacillus.* |
| C7 | *Limnohabitans planktonicus* ( LFYT01000006) 3-A5 | 99.47 | *Proteobacteria; Betaproteobacteria; Burkholderiales; Comamonadaceae; Limnohabitans.* |
| D1 | *Deinococcus antarcticus* (KC494323.1) | 100 | *Deinococcus-Thermus; Deinococci; Deinococcales; Deinococcaceae; Deinococcus.* |
| D2 | *Acidovorax temperans* ( KX622787.1) | 100 | *Proteobacteria; Betaproteobacteria; Burkholderiales; Comamonadaceae; Acidovorax.* |
| D3 | *Acidovorax caeni* (KJ806476.1) | 99.93 | *Proteobacteria; Betaproteobacteria; Burkholderiales; Comamonadaceae; Acidovorax.* |
| D4 | *Fictibacillus* sp. (MT541004.1) | 100 | *Firmicutes; Bacilli; Bacillales; Bacillaceae;Fictibacillus; unclassified Fictibacillus.* |
| D5 | *Bacillus cereus* (MT337533.1) | 100 | *Firmicutes; Bacilli; Bacillales; Bacillaceae; Bacillus;Bacillus cereus group.* |
| D6 | *Bacillus altitudinis* ( MT598007.1) | 100 | *Firmicutes; Bacilli; Bacillales; Bacillaceae; Bacillus.* |
| D7 | *Streptomyces albogriseolus*(MN658354.1) | 99.85 | *Actinobacteria; Streptomycetales; Streptomycetaceae;Streptomyces.* |
| D8 | *Paenibacillus glycanilyticus*(NR_024759.1) | 99.85 | *Firmicutes; Bacilli; Bacillales; Paenibacillaceae; Paenibacillus.* |
| D9 | *Exiguobacterium profundum* (NR_043204.1) | 99.79 | *Firmicutes; Bacilli; Bacillales; Bacillales Family XII.Incertae Sedis; Exiguobacterium.* |
| D10 | *Lysinibacillus sphaericus*(NR_112627.1) | 99.92 | *Firmicutes; Bacilli; Bacillales; Bacillaceae; Lysinibacillus.* |
| D11 | *Pseudomonas alcaligenes*(NR_114472.1) | 99.06 | *Proteobacteria; Gammaproteobacteria; Pseudomonadales; Pseudomonadaceae; Pseudomonas.* |
| D12 | *Micrococcus yunnanensis* (MT033093.1) | 100 | *Actinobacteria; Micrococcales; Micrococcaceae; Micrococcus.* |
| D13 | *Bacillus aerophilus*(MT102966.1) | 100 | *Firmicutes; Bacilli; Bacillales; Bacillaceae; Bacillus.* |
| D14 | *Bacillus stratosphericus*(MT071702.1) | 99.93 | *Firmicutes; Bacilli; Bacillales; Bacillaceae; Bacillus.* |
| D15 | *Pseudomonas alcaligenes*(MG438507.1) | 99.71 | *Proteobacteria; Gammaproteobacteria; Pseudomonadales;Pseudomonadaceae; Pseudomonas.* |
| D16 | *Bacillus pumilus*(MK757700.1) | 100 | *Firmicutes; Bacilli; Bacillales; Bacillaceae; Bacillus.* |
| D17 | *Acidovorax* sp. (MF370622.1) | 99.93 | *Proteobacteria; Betaproteobacteria; Burkholderiales; Comamonadaceae; Acidovorax.* |
| D18 | *Micromonospora* sp.(LC497886.1) | 100 | *Actinobacteria; Micromonosporales; Micromonosporaceae;Micromonospora.* |
| D19 | *Pseudomonas aeruginosa*(JF496542.1) | 99.35 | *Proteobacteria; Gammaproteobacteria; Pseudomonadales;Pseudomonadaceae; Pseudomonas.* |
| D20 | *Bacillus cereus* (GQ406846.1) | 99.59 | *Firmicutes; Bacilli; Bacillales; Bacillaceae; Bacillus;Bacillus cereus group.* |
| D21 | *Bacillus wiedmannii* (NR_152692.1) | 99.93 | *Firmicutes; Bacilli; Bacillales; Bacillaceae; Bacillus; Bacillus cereus group.* |
| D22 | *Cloacibacterium* sp.(LC094578.1) | 100 | *Bacteroidetes; Flavobacteriia; Flavobacteriales; Flavobacteriaceae.* |
| D23 | *Roseomonas* sp.(MK757949.1) | 100 | *Proteobacteria; Alphaproteobacteria; Rhodospirillales; Acetobacteraceae; Roseomonas.* |
| D24 | *Lysinibacillus fusiformis*(MF079273.1) | 100 | *Firmicutes; Bacilli; Bacillales; Bacillaceae; Lysinibacillus.* |
| D25 | *Pseudomonas alcaligenes* (MG438507.1) | 99.71 | *Proteobacteria; Gammaproteobacteria; Pseudomonadales; Pseudomonadaceae; Pseudomonas.* |
| D26 | *Bacillus thuringiensis*(MT052669.1) | 100 | *Firmicutes; Bacilli; Bacillales; Bacillaceae; Bacillus;Bacillus cereus group.* |
| D27 | *Microbacterium* sp. (MN410660.1) | 100 | *Actinobacteria; Micrococcales; Microbacteriaceae;Microbacterium.* |
| D28 | *Pseudomonas* sp. (DQ205301.1) | 99.72 | *Proteobacteria; Gammaproteobacteria; Pseudomonadales;Pseudomonadaceae; Pseudomonas.* |
| D29 | *Acidovorax* sp.(MF370622.1) | 99.86 | *Proteobacteria; Betaproteobacteria; Burkholderiales;Comamonadaceae; Acidovorax.* |
| D30 | *Micromonospora* sp. (MH780497.1) | 100 | *Actinobacteria; Micromonosporales; Micromonosporaceae; Micromonospora.* |
| D31 | *Micromonospora* sp. (LC497885.1) | 100 | *Actinobacteria; Micromonosporales; Micromonosporaceae; Micromonospora.* |
| D32 | *Micrococcus yunnanensis*(MT033093.1) | 100 | *Actinobacteria; Micrococcales; Micrococcaceae; Micrococcus.* |
| D33 | *Bacillus cereus* (MT020418.1) | 99.93 | *Firmicutes; Bacilli; Bacillales; Bacillaceae; Bacillus; Bacillus cereus group.* |
| D34 | *Chromobacterium violaceum*(NR_114954.1) | 100 | *Proteobacteria; Betaproteobacteria; Neisseriales; Chromobacteriaceae; Chromobacterium.* |
| D35 | *Rhodococcus* sp.(MN519546.1) | 100 | *Actinobacteria; Corynebacteriales; Nocardiaceae; Rhodococcus.* |
| D36 | *Streptomyces roseorubens*(MN558971.1) | 99.85 | *Actinobacteria; Streptomycetales; Streptomycetaceae; Streptomyces.* |
| D37 | *Acidovorax* sp.(MF370622.1) | 99.93 | *Proteobacteria; Betaproteobacteria; Burkholderiales; Comamonadaceae; Acidovorax.* |
| D38 | *Pseudomonas alcaligenes*(NR_114472.1) | 98.66 | *Proteobacteria;Gammaproteobacteria; Pseudomonadales;Pseudomonadaceae; Pseudomonas.* |
| D39 | *Micrococcus yunnanensis*(MT033093.1) | 100 | *Actinobacteria; Micrococcales; Micrococcaceae; Micrococcus.* |
| D40 | *Aeromonas enteropelogenes*(FJ940843.1) | 99.64 | *Proteobacteria; Gammaproteobacteria; Aeromonadales;Aeromonadaceae; Aeromonas.* |
| D41 | *Staphylococcus haemolyticus* (MT539735.1) | 100 | *Firmicutes; Bacilli; Bacillales; Staphylococcaceae; Staphylococcus.* |
| D42 | *Escherichia coli*(MN006360.1) | 99.79 | *Proteobacteria; Gammaproteobacteria; Enterobacterales; Enterobacteriaceae; Escherichia.* |
| D43 | *Macromonas* sp.(KU360711.1) | 98.31 | *Proteobacteria; Betaproteobacteria; Burkholderiales; Comamonadaceae; Malikia.* |
| D44 | *Pseudomonas otitidis*(NR_043289) | 99.93 | *Proteobacteria; Gammaproteobacteria; Pseudomonadales;Pseudomonadaceae; Pseudomonas.* |
| D45 | *Chromobacterium violaceum* (NR_114954.1) | 100 | *Proteobacteria; Betaproteobacteria; Neisseriales; Chromobacteriaceae; Chromobacterium.* |
| D46 | *Bacillus cereus*(NR_074540.1) | 99.93 | *Firmicutes; Bacilli; Bacillales; Bacillaceae; Bacillus; Bacillus cereus group.* |
| D47 | *Acidovorax caeni*(NR_042427.1) | 100 | *Proteobacteria; Betaproteobacteria; Burkholderiales;Comamonadaceae; Acidovorax.* |
| E1 | *Paenibacillus alvei*(NR_113577.1) | 99.01 | *Firmicutes; Bacilli; Bacillales; Paenibacillaceae; Paenibacillus.* |
| E2 | *Staphylococcus haemolyticus*(NR_025922.1) | 99.86 | *Firmicutes; Bacilli; Bacillales; Staphylococcaceae;Staphylococcus.* |
| E3 | *Cellulomonas* (MN371286.1) | 100 | *Actinobacteria; Micrococcales; Cellulomonadaceae; Cellulomonas.* |
| E4 | *Acidovorax* sp.(MF370622.1) | 99.93 | *Proteobacteria; Betaproteobacteria; Burkholderiales; Comamonadaceae; Acidovorax.* |
| E5 | *Aeromonas rivipollensis*(NR_144574.1 ) | 99.36 | *Proteobacteria; Gammaproteobacteria; Aeromonadales; Aeromonadaceae; Aeromonas.* |
| E6 | *Micrococcus yunnanensis* (NR_116578.1) | 99.71 | *Actinobacteria; Micrococcales; Micrococcaceae; Micrococcus.* |
| E7 | *Rhodococcus hoagii*(NR_116691.1) | 99.93 | *Actinobacteria; Corynebacteriales; Nocardiaceae;Rhodococcus.* |
| E8 | *Streptomyces acrimycini*(NR_112252.1 ) | 98.61 | *Actinobacteria; Streptomycetales; Streptomycetaceae; Streptomyces.* |
| E9 | *Porphyrobacter tepidarius*(NR_114652.1) | 99.78 | *Proteobacteria; Alphaproteobacteria; Sphingomonadales;Erythrobacteraceae; Porphyrobacter.* |
| E10 | *Novosphingobium* (NR_133800.1) | 99.26 | *Proteobacteria; Alphaproteobacteria; Sphingomonadales; Sphingomonadaceae; Novosphingobium.* |
| F1 | *Microbacterium schleiferi* (NR_044936.1) | 99.27 | *Actinobacteria; Micrococcales; Microbacteriaceae; Microbacterium.* |
| F2 | *Stenotrophomonas maltophilia*(CP051467.1) | 100 | *Proteobacteria;Gammaproteobacteria;Xanthomonadales;Xanthomonadaceae;Stenotrophomonas;Stenotrophomonas;* |
| G1 | *Staphylococcus warneri* (MT072204.1) | 100 | *Firmicutes; Bacilli; Bacillales; Staphylococcaceae; Staphylococcus.* |
| G2 | *Bacillus amyloliquefaciens* (MT613661.1) | 100 | *Firmicutes; Bacilli; Bacillales; Bacillaceae; Bacillus;Bacillus amyloliquefaciens group.* |
| G3 | *Staphylococcus hominis* MT072199.1 | 100 | *Firmicutes; Bacilli; Bacillales; Staphylococcaceae; Staphylococcus.* |
| G4 | *Bacillus haynesii*(NR_157609.1) | 99.65 | *Firmicutes; Bacilli; Bacillales; Bacillaceae; Bacillus.* |
| G5 | *Kocuria rhizophila*(NR_026452.1) | 99.86 | *Actinobacteria; Micrococcales; Micrococcaceae; Kocuria.* |

Table S7. Number of isolates from DWTS summarized at the genus level.

| Phylum | Class | Genus |  |  |  |  |  |  |  |  |
| --- | --- | --- | --- | --- | --- | --- | --- | --- | --- | --- |
|  |  |  | A | B | C | D | E | F | G | Total |
| *Proteobacteria* | *α-Proteobacteria* | *Porphyrobacter* |  |  |  |  | 1 |  |  | 1 |
|  |  | *Novosphingobium* |  |  |  |  | 1 |  |  | 1 |
|  |  | *Roseomonas* |  |  |  | 1 |  |  |  | 1 |
|  | *β-Proteobacteria* | *Hydrogenophaga* |  | 5 |  |  |  |  |  | 5 |
|  |  | *Acidovorax* |  | 1 |  | 6 | 1 |  |  | 8 |
|  |  | *Chromobacterium* |  | 2 | 1 | 2 |  |  |  | 5 |
|  |  | *Malikia* |  |  |  | 1 |  |  |  | 1 |
|  | *Gammaproteobacteria* | *Dickeya* | 1 |  |  |  |  |  |  | 1 |
|  |  | *Pseudomonas* |  | 3 |  | 7 |  |  |  | 10 |
|  |  | *Enterobacter* |  | 1 |  |  |  |  |  | 1 |
|  |  | *Escherichia* |  |  |  | 1 |  |  |  | 1 |
|  |  | *Aeromonas* |  | 1 |  | 1 | 1 |  |  | 3 |
|  |  | *Moraxella* |  | 1 |  |  |  |  |  | 1 |
| *Actinobacteria* |  | *Tessaracoccus* | 1 |  |  |  |  |  |  | 1 |
|  |  | *Streptomyces* |  |  |  | 2 | 1 |  |  | 3 |
|  |  | *Micrococcus* |  |  |  | 3 | 1 |  |  | 4 |
|  |  | *Micromonospora* |  |  |  | 3 |  |  |  | 3 |
|  |  | *Microbacterium* |  |  |  | 1 |  | 1 |  | 2 |
|  |  | *Rhodococcus* |  |  |  | 1 | 1 |  |  | 2 |
|  |  | *Cellulomonas* |  |  |  |  | 1 |  |  | 1 |
|  |  | *Kocuria* |  |  |  |  |  |  | 1 | 1 |
| *Firmicutes* |  | *Bacillus* | 1 | 3 | 3 | 10 |  |  | 2 | 19 |
|  |  | *Staphylococcus* |  |  | 1 | 1 | 1 |  | 2 | 5 |
|  |  | *Priestia* | 1 |  |  |  |  |  |  | 1 |
|  |  | *Paenibacillus* | 1 |  | 1 | 1 | 1 |  |  | 4 |
|  |  | *Fictibacillus* | 1 | 1 |  | 1 |  |  |  | 3 |
|  |  | *Exiguobacterium* |  |  |  | 1 |  |  |  | 1 |
|  |  | *Lysinibacillus* |  |  |  | 2 |  |  |  | 2 |
| *Bacteroidetes* |  | *Flavobacteriaceae* |  |  |  | 1 |  |  |  | 1 |
| *Deinococcus-Thermus* |  | *Deinococcus* |  |  |  | 1 |  |  |  | 1 |
| Total |  |  | 6 | 18 | 6 | 47 | 10 | 1 | 5 | 93 |

Abbreviations: A, raw water; B:grid reaction tank effluent; C: settling pond effluent; E: sand filter effluent F: finished water; G: tap water.
